# Non‐Line‐of‐Sight Passive Ammonia Sensor Loaded With MXene/In_2_O_3_ Composites for Agricultural Products Quality Deterioration Detection

**DOI:** 10.1002/advs.76454

**Published:** 2026-07-03

**Authors:** Guoping Hu, Lin He, Fanli Meng, Daming Dong, Guolong Shi

**Affiliations:** ^1^ School of Artificial Intelligence Anhui Agricultural University Hefei Anhui China; ^2^ School of Information Science and Engineering Northeastern University Shenyang China; ^3^ Intelligent Equipment Research Center Beijing Academy of Agriculture and Forestry Sciences Beijing China; ^4^ Key Laboratory of Agricultural Sensors Ministry of Agriculture and Rural Hefei China

**Keywords:** agricultural product quality monitoring, ammonia sensor, dielectric transmission loss, non‐line‐of‐sight passive detection, RFID technology

## Abstract

Passive radio‐frequency identification technology (RFID) shows potential for non‐line‐of‐sight (NLoS) sensing, but electromagnetic attenuation and reflection losses caused by dielectric absorption and multipath effects reduce detection accuracy and stability. In this work, a flexible passive RFID sensor for NLoS ammonia detection is developed by integrating a spiral antenna with an LC interdigital electrode. MXene is modified with In_2_O_3_ nanoparticles to form a gas‐sensitive layer with enhanced surface activity and electronic modulation, thereby strengthening the ammonia‐induced frequency response. Moreover, structural separation of electromagnetic coupling and sensing functions mitigates the nonuniform distribution of coupled electromagnetic energy in the sensing tag. The experimental results demonstrate response characteristics under NLoS dielectric interference, with an *S*_11_ amplitude attenuation of *Δ|S*_11_ *| *= 9.19 dB and an ammonia‐induced resonance frequency shift of *Δ*|*f*| = 0.48 MHz. On this basis, an *S*_11_ amplitude‐frequency signal separation strategy is innovatively proposed to decouple NLoS dielectric interference signals from gas‐induced response signals, thereby clarifying the frequency perturbation effect of dielectric interference on ammonia sensing signals. Combined with electromagnetically decoupled signals, dielectric interference compensation is performed under conditions of 25°C and 40% RH. NLoS dielectric compensation stabilizes ammonia‐induced frequency shifts, suppresses interference, and enables reliable, rapid, nondestructive detection of quality deterioration in sealed fresh‐food packaging.

## Graphical abstract

This study presents a flexible, battery‐free passive RFID sensing platform for NLoS monitoring of quality deterioration in fresh agricultural products. It utilizes a structurally decoupled spiral antenna‐LC electrode architecture to suppress electromagnetic interference caused by packaging. Additionally, an RF signal decoupling paradigm is introduced to effectively isolate gas signals from medium‐induced interference, improving sensing accuracy and stability in sealed packaging environments.

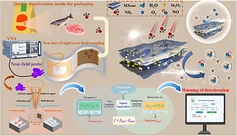

## 1 Introduction

Food safety issues arising from the deterioration of the quality of agricultural products have become increasingly severe. Consequently, achieving real‐time monitoring and early warning of safety during the storage and transportation of agricultural products has emerged as an urgent challenge [1, 2, 3]. The use of intelligent gas sensors for agricultural product quality assessment enables the early identification of spoilage or deterioration signals, thereby offering considerable research value and practical relevance. Ammonia is recognized as a characteristic marker gas released when the quality of fresh agricultural products deteriorates, and its concentration variations are closely correlated with the quality status of the products [4, 5, 6]. Various analytical methods, including chromatographic techniques [7], spectroscopic methods [8], and conventional chemical gas sensing technologies [9, 10], have been applied to monitor and analyze ammonia during the spoilage process of agricultural products. However, most of these approaches are typically applicable under line‐of‐sight detection conditions and often rely on destructive operations involving the packaging structure or the agricultural products themselves. Consequently, they are not well suited for rapid, nondestructive ammonia detection under non‐line‐of‐sight conditions during the storage, transportation, or packaging of agricultural products [11].

With the rapid advancement of radio‐frequency (RF) sensing technology and flexible electronics, flexible chipless RFID sensors have emerged as a promising approach for enabling non contact and non destructive detection of agricultural product quality [12, 13]. Owing to their chipless and passive characteristics, such sensors can achieve contactless detection of internal environmental parameters within packaging by monitoring the electromagnetic coupling between the reader antenna and the sensing tag, without compromising the integrity of the agricultural product packaging. Moreover, wireless transmission of RF signals is realized through an interrogation antenna, enabling passive perception of the internal packaging environment [14, 15]. Among the various sensing configurations, LC‐based sensing structures have attracted considerable attention because of their excellent frequency selectivity and resonance characteristics. These features allow LC resonators to respond sensitively to variations in environmental parameters that induce changes in dielectric permittivity or electrical conductivity, thereby demonstrating considerable potential for real‐time environmental monitoring applications [16, 17, 18, 19]. However, when LC structures are simultaneously employed for target sensing and signal transmission, issues such as signal crosstalk and nonuniform energy coupling are likely to arise, degrading the readout accuracy of the sensor system [20, 21]. In addition, gas‐sensitive materials, as critical components of the sensing structure, play a decisive role in determining the detection efficiency of RFID‐based sensors [22, 23, 24]. Among the various candidates, MXenes have attracted considerable attention due to their unique physicochemical properties. As an emerging class of two‐dimensional materials, these materials exhibit a high specific surface area and excellent electrical conductivity, along with tunable chemical compositions, layered structures, and abundant surface functional groups, making them an ideal platform for constructing high‐performance nanocomposite sensing materials [25, 26, 27]. Nevertheless, gas sensors based on pristine MXenes still suffer from limitations such as low response and baseline drift, which hinder their stability and reliability in practical applications [28, 29, 30]. Therefore, MXene‐based materials to enhance gas‐sensing performance and suppress baseline drift are highly important for improving their application potential in flexible passive RFID gas sensing systems.

Currently, research on the passive detection of quality deterioration in fresh agricultural products has achieved notable progress [31, 32]. However, existing studies are still predominantly conducted under ideal line‐of‐sight (LoS) conditions, with insufficient attention given to the non‐line‐of‐sight (NLoS) environments that are ubiquitous in practical agricultural product monitoring scenarios [33]. In particular, the effects of complex media such as packaging materials on RF signal propagation—including transmission loss, reflection‐induced interference, and their coupling mechanisms with gas sensing signals—remain inadequately investigated. Under NLoS conditions for passive RF sensing, electromagnetic waves are generally required to propagate through agricultural product packaging media via transmission paths in the absence of a direct propagation link. Variations in the dielectric properties of packaging materials introduce reflection loss and absorption loss during propagation, considerably increasing electromagnetic energy dissipation and leading to pronounced attenuation of RF signals [34, 35]. Furthermore, as environmental humidity increases, continuous or discretely distributed molecular water films may form on the surface of packaging materials, thereby altering the overall electromagnetic properties of the medium and affecting the reflection behavior of electromagnetic waves at material interfaces [36]. Moreover, an increase in the thickness of the packaging medium extends the propagation path length of electromagnetic waves within the material, resulting in a cumulative increase in absorption effects [37, 38]. Ultimately, the electromagnetic energy transmitted through the packaging medium is substantially attenuated, leading to variations in the amplitude of the sensor echo signal. When the energy loss exceeds a certain threshold, frequency drift of the RF resonance peaks may occur, causing a shift in the sensor's gas response characteristics and consequently degrading the accuracy and stability of gas detection.

To address the aforementioned challenges, this work proposes a sensitive‐material‐loaded flexible passive sensing scheme for NLoS detection of quality deterioration in fresh agricultural products. First, a flexible chipless RFID sensing tag was fabricated on a flexible polyimide substrate by using the screen‐printing technique. The proposed sensing tag adopts a spiral coil antenna as the signal transmission module and an LC interdigital capacitor as the sensing module, thereby achieving a structured separation between signal transmission and sensing functions. A gas‐sensitive layer was constructed by decorating MXenes with In_2_O_3_ nanoparticles, which increased the number of active sites and electronic modulation effects, thereby improving the sensing response toward volatile ammonia released when the quality of fresh agricultural products deteriorates. The transmission loss characteristics of electromagnetic waves propagating through packaging media under non‐line‐of‐sight conditions, as well as the RF interrogation response variations induced by ammonia adsorption, which were quantitatively characterized. The results indicate that transmission loss introduced by packaging media primarily leads to attenuation in the amplitude of the *S*_11_ signal, whereas ammonia adsorption predominantly causes frequency shifts. On the basis of these observations, an innovative *S*_11_ amplitude‐frequency signal decoupling strategy is proposed, enabling effective separation of medium‐induced interference effects and gas‐sensing responses. Furthermore, a correlation mechanism between medium transmission loss and *S*_11_ amplitude variation is established. By combining the frequency perturbation characteristics induced by transmission loss under different medium conditions, computational compensation of RF signals affected by non‐line‐of‐sight medium interference is achieved. Finally, the proposed technique is applied to an intelligent agricultural product packaging scenario to realize the passive detection of quality deterioration in fresh agricultural products under NLoS packaging conditions. This work validates the application potential of the proposed method for intelligent agricultural product monitoring and lay a foundation for future studies that integrate algorithm‐based RF signal feature extraction and quality prediction.

## 2 Results and Discussion

### 2.1 Design and Simulation Analysis of the Passive Sensing Tag

As shown in Figure 1a, the proposed chipless RFID sensing tag is fabricated on a flexible polyimide (PI) substrate, enabling conformal attachment to packaging surfaces, thereby meeting the practical requirements of real‐world packaging applications.

**FIGURE 1:**
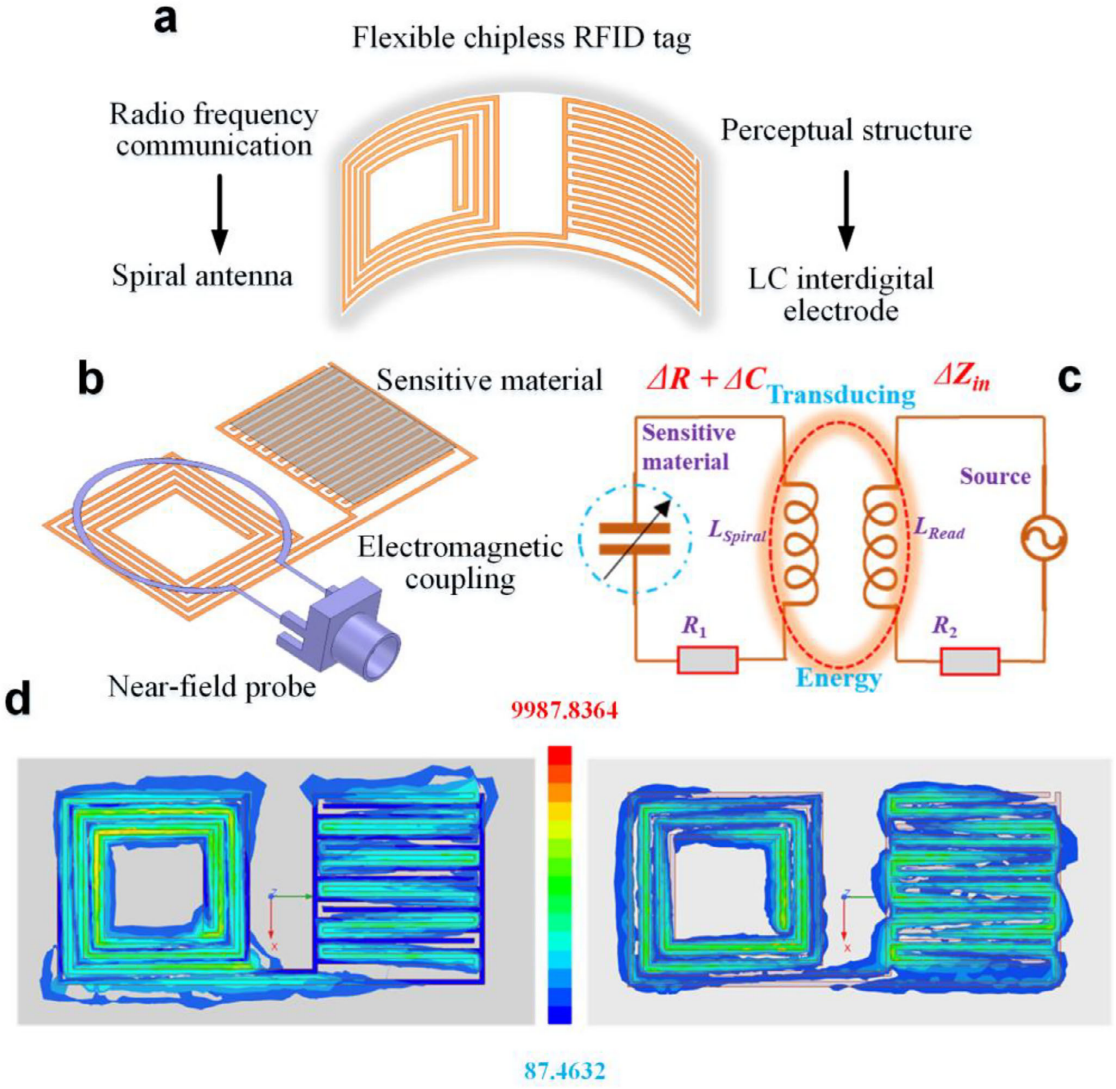
(a) Flexible chipless RFID sensing tag, (b) Simulation analysis of the passive sensing process, (c) Equivalent circuit model for passive sensing, (d) Electric‐field energy distribution of the electromagnetically coupled sensing tag.

To investigate and optimize the electromagnetic performance of the sensing tag, full‐wave electromagnetic simulations were conducted using the high‐frequency electromagnetic simulation software HFSS. By optimizing the geometric parameters of the tag, favorable impedance matching was achieved, which effectively enhances the energy coupling efficiency and signal transmission performance (Figures S1, S2). In order to elucidate the passive sensing mechanism based on the electromagnetic coupling detection scheme between the near‐field probe and the sensing tag, the passive sensing process of the chipless RFID sensor was analyzed through electromagnetic simulations (Figure 1b). The corresponding equivalent passive sensing circuit is illustrated in Figure 1c.Unlike conventional LC‐based designs, the sensing and signal transmission functions are highly coupled and simultaneously implemented within a single resonant structure. This work proposes a structure by integrating a spiral coil antenna with an LC interdigital electrode; the proposed architecture effectively mitigates electromagnetic energy non‐uniformity induced by functional coupling, thereby improving sensing stability and signal readout reliability (Figure 1d).

### 2.2 Characterization of MXene/in_2_O_3_ Composites

The morphology and microstructure of the composite were examined by scanning electron microscopy (SEM) and transmission electron microscopy (TEM). The SEM images reveal that the MXene sheets exhibit a typical layered structure, while In_2_O_3_ nanoparticles are uniformly distributed and embedded on the surface of the two‐dimensional MXene sheets, forming a dense composite interface (Figure 2a,b). This structural configuration not only effectively suppresses the oxidation of MXenes in air but also provides efficient pathways for charge transport and interfacial reactions [39, 40]. TEM images and the corresponding elemental mapping results further confirm the compositional characteristics of the composite system. Specifically, C is distributed mainly within the MXene matrix, whereas In and O are uniformly dispersed on the MXene sheet surfaces and within the porous regions, demonstrating that In_2_O_3_ nanoparticles are well dispersed and firmly anchored throughout the two‐dimensional substrate (Figure 2c).

**FIGURE 2:**
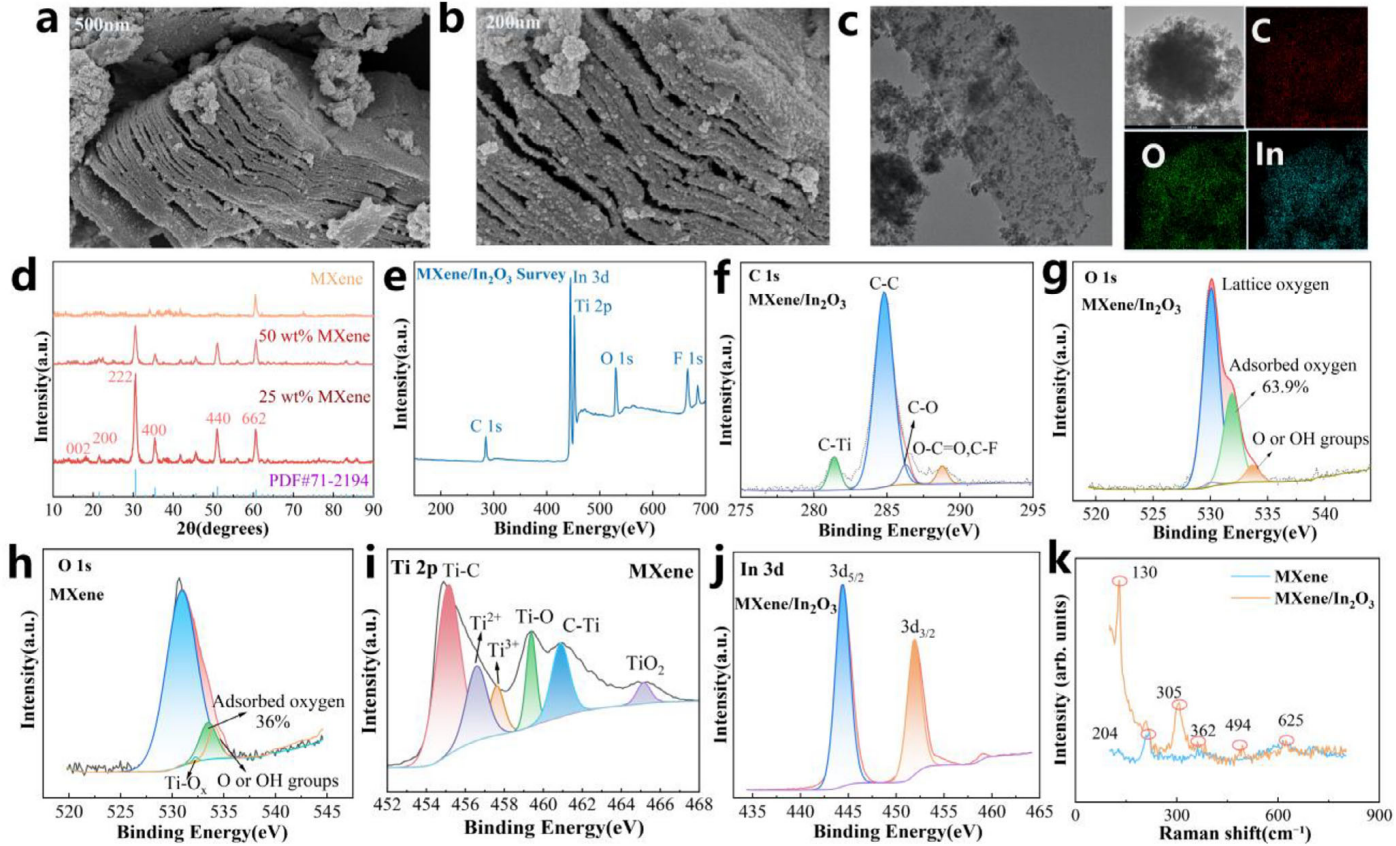
Characterization of MXene/In_2_O_3_: SEM images of MXene/In_2_O_3_ at different magnifications: (a) 500 nm and (b) 200 nm, (c) EDS mapping of MXene/In_2_O_3_, (d) X‐ray diffractions of MXene and MXene/In_2_O_3_ hybrids, XPS spectra of MXene/In_2_O_3_: (e) Survey, (f) C 1s, (g) O 1s, and (j) In 3d, XPS spectrum of MXene: (h) O 1s, and (i) Ti 2p; (k) Raman spectra of MXene and MXene/In_2_O_3_ materials.

Additionally, the x‐ray diffraction (XRD) pattern of the MXene/In_2_O_3_ composite exhibits distinct diffraction peaks at 2θ values of 18.2°, 21.5°, 30.5°, 35.3°, 50.9°, and 60.5°, which can be indexed to the (200), (211), (222), (400), (440), and (622) crystal planes of In_2_O_3_, respectively (JCPDS No. 06–0416). The presence of these characteristic diffraction peaks indicates that In_2_O_3_ retains good crystallinity and a typical cubic phase structure within the composite system. Moreover, compared with that of pristine MXene, the (002) diffraction peak of the MXene in the composite exhibits a noticeable shift in position, accompanied by changes in peak intensity. This behavior can be attributed to interfacial interactions and stress modulation effects between the In_2_O_3_ nanoparticles and the MXene layers, confirming the formation of an effective coupled structure at the microscopic scale (Figure 2d).

To further elucidate the elemental composition and chemical bonding states of the composite, x‐ray photoelectron spectroscopy (XPS) measurements were performed on the MXene/In_2_O_3_ composite system. As shown in Figure 2e, the survey spectrum reveals that the composite primarily comprises C, In, Ti, O, and F. The high‐resolution C 1s spectrum (Figure 2f) can be deconvoluted into four characteristic peaks located at 281.3 eV (C─Ti), 284.7 eV (C─C), 286.2 eV (C─O), and 288.7 eV (O─C─O and C─F), confirming that the surface functional groups and the intrinsic framework of the MXene are well preserved. The O 1s XPS spectra of the MXene/In_2_O_3_ composite and pristine MXene are shown in Figure 2g,h, respectively. For MXene, the O 1s spectrum can be fitted with three peaks at 532.28, 533.58, and 534.08 eV, which are attributed to Ti─O_x_ bonds, chemisorbed oxygen, and surface hydroxyl groups (─O/─OH), respectively. In contrast, the O 1s spectrum of the MXene/In_2_O_3_ composite presents three peaks, corresponding to lattice oxygen originating from In_2_O_3_, adsorbed oxygen species, and surface hydroxyl groups (─O/─OH), respectively. Notably, the appearance of the lattice oxygen peak provides direct evidence for the successful incorporation and immobilization of In_2_O_3_ within the MXene matrix. Moreover, compared with that of pristine MXene, the relative content of adsorbed oxygen in the MXene/In_2_O_3_ composite increases significantly from 36% to 63.9%. This enhancement offers more available active sites for the generation of reactive oxygen species, thereby effectively improving the gas‐sensing performance of the composite material. In the high‐resolution Ti 2p spectrum (Figure 2i), the peaks of Ti2p^3/2^ are centralized at 455.18, 456.58, 457.68, and 459.38 eV, corresponding to Ti─C, Ti^2+^ (Ti─X), Ti^3+^(Ti_x_O_y_), and Ti^4+^ (Ti─O). The fitted peaks of Ti 2p^1/2^ appear at 461.88 eV (C─Ti) and 465.18 eV (TiO_2_). Additionally, as shown in Figure 2j, the introduction of In_2_O_3_ results in two characteristic peaks of In^3+^ located at 444.3 eV (In 3d^5/2^) and 451.8 eV (In 3d^3/2^). These XPS results collectively indicate the formation of a stable heterointerface between MXene and In_2_O_3_.

Comparative analysis of the Raman spectra in the fingerprint region ranging from 100 to 800 cm^−1^ further revealed the interfacial coupling characteristics of the composite system (Figure 2k). Pristine MXenes exhibit a characteristic peak at 204 cm^−1^, which is assigned to the A_1_g out‐of‐plane vibrational mode of the Ti─C bond. In contrast, this dominant peak completely disappears for the MXene/In_2_O_3_ composite, accompanied by the emergence of a full set of characteristic Raman peaks of cubic‐phase In_2_O_3_ at 130, 305, 362, 494, and 625 cm^−1^. The Raman bands exhibit pronounced broadening and partial overlap, indicating the presence of strong electronic coupling effects between the In_2_O_3_ nanoparticles and the MXene layers rather than simple physical mixing. To sum up, the multidimensional characterization results collectively confirm that In_2_O_3_ nanoparticles are successfully and uniformly anchored onto the layered MXene substrate, resulting in significant interfacial stress modulation and electronic coupling effects. Such synergistic interfacial interactions provide enhanced mechanisms for gas adsorption, charge transport, and subsequent sensing responses.

### 2.3 Gas Sensing Mechanism

As illustrated in Figure 3a, passive RF sensing under NLoS conditions can be applied to the detection of fresh agricultural products in sealed packaging. Owing to the absence of a direct LoS propagation path between the near‐field probe and the flexible chipless sensing tag, RF signals are required to propagate through the agricultural product packaging medium via transmission. Under NLoS conditions, the RF signal propagates through the packaging medium and couples electromagnetically with the flexible chipless RFID sensing tag. The spiral coil antenna of the tag is excited via electromagnetic induction, harvesting energy from the incident magnetic field and transferring it to the connected interdigital electrode structure. The spiral coil antenna and interdigital electrode constitute an LC resonant circuit, in which the coil antenna provides the equivalent inductance *L*, while the interdigital electrode loaded with the gas‐sensitive material contributes the equivalent capacitance (*C*_IDE_).

**FIGURE 3:**
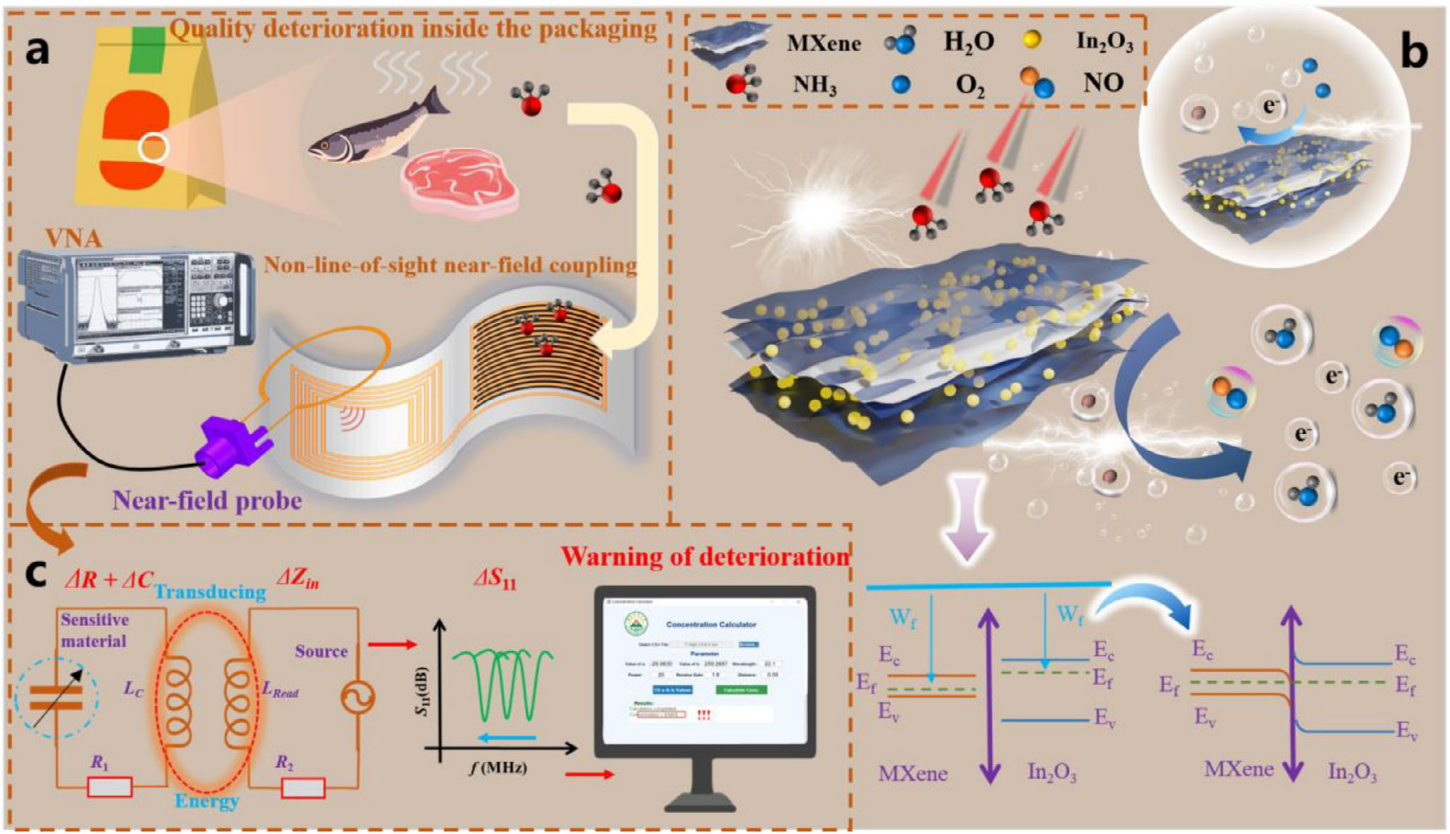
(a) Schematic illustration of the passive detection of fresh agricultural product quality deterioration under NLoS conditions, (b) Sensing mechanism of ammonia adsorption on MXene/In_2_O_3_ composite materials, and (c) Equivalent circuit model of the NLoS passive RF sensing system.

In order to achieve efficient ammonia sensing, In_2_O_3_ nanoparticles were incorporated into the MXene matrix in this work. Owing to its high carrier concentration, Ti_3_C_2_T_x_ MXene exhibits metallic characteristics and consequently shows a relatively weak response to NH_3_. After compositing MXene with In_2_O_3_ nanoparticles, a p–n heterojunction is formed at the interface [41]. The gas sensing mechanism is primarily attributed to the changes in electrical response induced by the adsorption and desorption processes. Under room‐temperature conditions, oxygen molecules adsorbed on the surface capture electrons [42, 43], leading to the formation of ionized adsorbed oxygen species (O_2_^−^):

(1) $$O_{2\left(\mathrm{gas}\right)}+e^{-}\to O_{2\left(\mathrm{ads}\right)}^{-}$$

The adsorption of oxygen species leads to a reduction in carrier concentration, resulting in the formation of a depletion layer on the particle surface. This process narrows the conductive pathways and drives the material into a high‐resistance state. Meanwhile, the presence of the depletion layer weakens the effective dielectric constant of the sensing material between the interdigital electrodes, thereby causing a decrease of *C*_IDE_. In addition, since the Fermi level of n‐type In_2_O_3_ is higher than that of p‐type MXene, electrons transfer from In_2_O_3_ to MXene at the interface until Fermi‐level equilibrium is established. During this process, a pronounced electron depletion layer is formed on the In_2_O_3_ side, and a stable Schottky barrier is established at the heterointerface.

(2) $$NH_{3\left(\mathrm{gas}\right)}\to NH_{3\left(\mathrm{ads}\right)}$$

(3) $$4NH_{3\left(\mathrm{ads}\right)}+5{O^{-}}_{2\left(\mathrm{ads}\right)}\to 4NO_{\left(\mathrm{gas}\right)}+6H_{2}O_{\left(\mathrm{gas}\right)}+10e^{-}$$

Upon exposure to ammonia, adsorbed O_2_^−^ species on the surface react with NH_3_ molecules, producing nitrogen oxides (e.g., NO_x_) and releasing trapped electrons. The released electrons subsequently recombine with the holes, reducing the concentration in the dominant conduction channels. Consequently, the equivalent resistance (*R*) of the interdigital electrode decreases. During this process, the depletion layer in In_2_O_3_ reduced and the interfacial barrier declined, which facilitated carrier transport across the junction. Consequently, the conductive pathways are recovered, leading to a transition from a high‐resistance to a low‐resistance state (Figure 3b).

According to the equivalent circuit of passive sensing (Figure 3c), the adsorption of NH_3_ by the MXene/In_2_O_3_ composite leads to increases in *C*_NH3_ of the interdigital electrode while modulating the impedance *Z*_LC_ of the composite material (Equations (4) and (5)). This impedance modulation disrupts the original impedance matching between the interdigital electrode and the spiral coil antenna, which reduces energy transfer efficiency between the near‐field probe and the sensing tag. Consequently, NH_3_ concentration is transduced into measurable resonance frequency shifts in the RF response [44, 45] (Equation (6)), enabling fully passive and noncontact ammonia detection under NLoS packaging conditions.

(4) $$C_{\mathrm{IDE}}=C_{0}+\Delta C_{NH_{3}}$$

(5) $$Z_{\mathrm{LC}}=R+j\omega L+\frac{1}{j\omega C_{\mathrm{IDE}}}$$

(6) $$f=\frac{1}{2\pi \sqrt{LC_{\mathrm{IDE}}}}$$

where *f* denotes the resonant frequency, *C*_0_ represents the initial capacitance of the interdigitated electrode loaded with the gas‐sensitive material, *ΔC*_NH3_ corresponds to the increase in capacitance induced by NH_3_ adsorption, *R* is the equivalent resistance, and *ω* denotes the angular frequency.

### 2.4 Sensing Performance of the MXene/In_2_O_3_‐loaded Flexible Passive Ammonia Sensor

To evaluate the ammonia sensing performance of the MXene/In_2_O_3_ composite, comparative experiments were performed using the laboratory ammonia testing system shown in Figure S3. Flexible chipless RFID ammonia sensors loaded with pristine MXene and MXene/In_2_O_3_ composites were evaluated over an NH_3_ concentration range of 3–20 ppm, and their RF responses after coupling with a near‐field probe were analyzed. As shown in Figure S4a,b, the resonant frequencies of both sensors exhibited pronounced shifts with increasing NH_3_ concentration. Notably, the MXene‐based sensor showed a frequency shift of approximately 380 kHz with a sensitivity of 22.35 kHz/ppm, whereas the MXene/In_2_O_3_ sensor showed a shift of approximately 480 kHz with a sensitivity of 28.23 kHz/ppm, corresponding to a 26.3% increase in sensitivity (Figure 4a). As illustrated in Figure 4b, both sensors exhibit a good linear relationship between the resonance frequency shift and the NH_3_ concentration in the range of 3–20 ppm. The linear fitting results further confirm the stable and reliable gas‐sensing characteristics of the proposed sensors.

**FIGURE 4:**
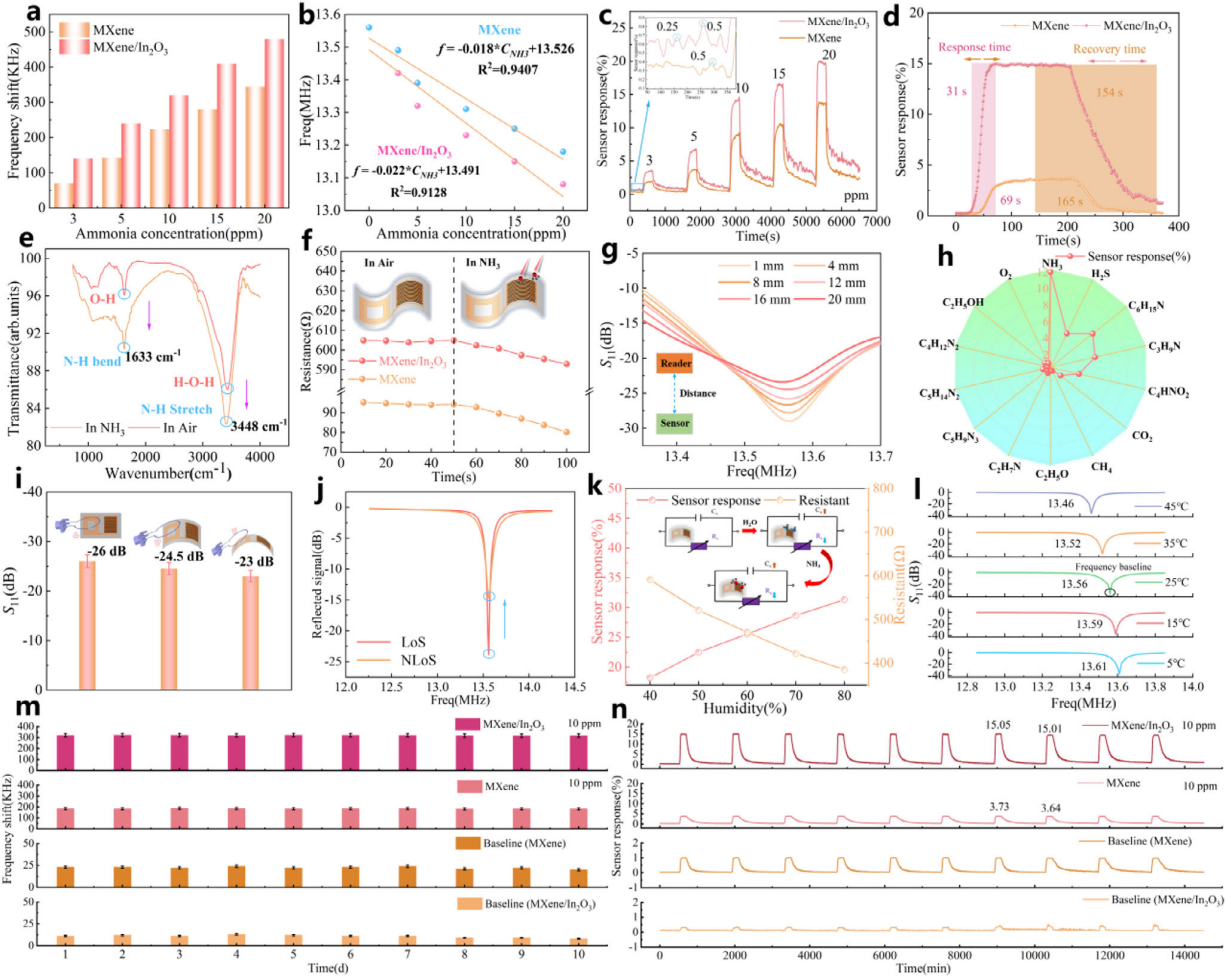
Ammonia sensing performance of the flexible chipless RFID sensor: (a) Frequency response of the MXene and MXene/In_2_O_3_ sensors under different ammonia concentrations, (b) Fitted frequency shift response curve versus NH_3_ concentration, (c) Dynamic response curve of ammonia adsorption, (d) Response and recovery time curve for ammonia adsorption, (e) FTIR spectra of the MXene/In_2_O_3_ composite before and after NH_3_ adsorption, (f) Resistance changes of the MXene material sensors and the MXene/In_2_O_3_ material sensors after ammonia adsorption, (g) *S*‐parameter response at various coupling distances, (h) Gas selectivity response of the sensor, (i) The *S*_11_ signals of the flexible tag under different bending states, (j) Reflection signal strength between LoS and NLoS conditions, (k) The sensing response affected by humidity, (l) Baseline drift curve of temperature fluctuation sensing, Long‐term stability test: (m) Frequency shift, and (n) Sensing response to ammonia adsorption.

To determine the practical detection limit of the MXene/In_2_O_3_‐loaded passive RFID sensor, its dynamic response was evaluated under different NH_3_ concentrations (Figure 4c). The sensor response exhibited a monotonic increase with increasing NH_3_ concentration, demonstrating stable and repeatable concentration‐dependent behavior. Meanwhile, the MXene/In_2_O_3_ composite sensor exhibited a significantly enhanced response toward ammonia compared with the pristine MXene sensor. At 250 ppb, the response approached the system background noise level, resulting in poor signal distinguishability. Upon increasing the concentration to 500 ppb, a stable and well‐resolved response peak emerged. Therefore, 500 ppb was defined as the practical detection limit, representing the lowest detectable concentration capable of generating a clear and reproducible signal above baseline noise. Owing to enhanced interfacial modulation, facilitated carrier transport, and increased active adsorption sites within the MXene/In_2_O_3_ composite, the sensor exhibited markedly improved sensitivity, thereby significantly lowering the detection limit. This detection capability satisfies the requirement for monitoring trace ammonia released during the early stages of fresh meat spoilage. In addition, the MXene/In_2_O_3_ composite material‐loaded passive sensor also shows significant advantages in both response and recovery times. The response time is approximately 31 s, markedly shorter than the 69 s of pure MXene, indicating that the composite material can more rapidly respond to ammonia adsorption. The recovery time is about 154 s, slightly better than the 165 s of pure MXene, demonstrating superior desorption kinetics (Figure 4d). (The calculation of the sensing response percentage is provided in Note SI.)

Fourier transform infrared (FTIR) spectroscopy was employed to compare the spectral features of the material before and after NH_3_ exposure. As shown in Figure 4e, a significantly intensified and broadened absorption band appeared at 3448 cm^−1^ after ammonia adsorption. This band originates from the coupled superposition of the N─H stretching vibrations of adsorbed NH_3_ molecules and the O─H stretching vibrations on the material surface. Moreover, the absorption peak at approximately 1633 cm^−1^, corresponding to the N─H bending vibration, sharply increased in intensity and was synergistically enhanced by the H─O─H bending vibration of surface‐adsorbed water molecules. These spectral changes indicate that NH_3_ molecules form coordination bonds with surface Lewis acid sites (In^3+^) via the lone pair electrons of nitrogen atoms, providing direct evidence for the chemisorption of ammonia on the composite surface. Furthermore, the resistance variation of the sensing tag before and after NH_3_ adsorption was measured using a digital source meter (Figure S5). As shown in Figure 4f, a significant decrease in resistance is observed upon NH_3_ adsorption. This electrical response is consistent with the chemisorption process revealed by FTIR analysis.

To investigate the influence of the separation distance between the near‐field probe and the flexible chipless RFID tag on the signal readout performance under near‐field coupling conditions, the variation in the signal response as a function of the probe‐tag distance is illustrated in Figure 4g. As the distance increases, the *S*_11_ value gradually increases due to the combined effects of the attenuation of the near‐field coupling energy and reduced signal transmission efficiency. In addition, selectivity tests were conducted using volatile basic nitrogen (TVB‐N) gases commonly generated during the spoilage of fresh meat products [46, 47]. The results indicate that, compared with NH_3_, other interfering gases induce only negligible sensing response percentages, demonstrating the excellent selectivity of the proposed sensor and its low cross‐sensitivity (Figure 4h). To assess the mechanical stability of the flexible chipless RFID sensor under practical operating conditions, its sensing performance was evaluated at different bending degrees (Figure 4i). The results show that mechanical bending slightly weakens the coupling effect of the sensing film, leading to minor shifts in the *S*_11_ response. However, the overall signal variation remains within an acceptable range, indicating good mechanical robustness and reliability of the flexible sensor.

Furthermore, the signal intensity of the flexible chipless RFID sensing tag under NLoS conditions was systematically investigated (Figure S6 and Videos S1, S2). Under an NLoS environment with the presence of obstacles, the signal amplitude experiences varying degrees of attenuation, which can be attributed primarily to electromagnetic wave absorption and reflection effects induced by the obstructing media (Figure 4j). As shown in Figure 4k, the sensor response to 10 ppm NH_3_ was evaluated under various relative humidity (RH) levels ranging from 40% to 80%. The experimental results show that the sensing response increases significantly with environmental humidity, rising from 17% to 32%. With increasing humidity, adsorbed water molecules facilitate the reaction between NH_3_ and surface oxygen species, releasing additional electrons. This enhances interfacial charge transfer, increases conductivity and capacitance, reduces resistance, and consequently amplifies the resonant frequency shift. (The mechanism underlying humidity‐induced baseline drift of the sensor is detailed in Note SII). In addition, baseline drift measurements were conducted over a temperature range of 5°C–45°C, which is typical for agricultural product storage and transportation. As shown in Figure 4l, when the temperature decreases to 5°C, the baseline resonant frequency increases by approximately 50 kHz relative to that at 25°C, whereas increasing the temperature to 45°C results in a decrease of about 80 kHz. Owing to temperature‐induced modulation of the carrier concentration, interfacial charge distribution, and polarization characteristics within the sensing layer, the sensor's overall resistance and equivalent dielectric parameters are altered, ultimately resulting in baseline drift of the resonant frequency. (The mechanism underlying temperature‐induced baseline drift of the sensor is detailed in Note SIII). These results indicate that variations in ambient temperature can induce significant baseline frequency drift, thereby affecting the accuracy of ammonia detection. In subsequent experiments and data processing, the influence of environmental factors on the sensor baseline was fully considered, and the frequency shifts induced by such factors were systematically corrected to ensure the reliability of the sensing results.

Finally, Figure 4m,n evaluate the long‐term stability of the sensors. Under ambient conditions (25°C and ≈40% relative humidity), the MXene/In_2_O_3_ composite sensor and the pristine MXene sensor were simultaneously exposed to air, while their RF response signals were periodically recorded. The MXene/In_2_O_3_ composite sensor exhibits only a slight drift of ≈10 kHz in the *S*_11_ resonance frequency baseline throughout the test period. In contrast, the pristine MXene sensor shows a substantially larger baseline drift of ≈23 kHz, accompanied by pronounced signal fluctuations, indicating inferior environmental stability. Furthermore, the long‐term sensing durability was evaluated under 10 ppm NH_3_. The MXene/In_2_O_3_ composite sensor maintains a pronounced frequency shift of ≈300 kHz throughout the entire testing period, indicating a stronger response capability toward NH_3_. Meanwhile, the corresponding sensing response remains stable at 15% ± 0.05%. By comparison, the pristine MXene sensor exhibits only a frequency shift of ≈180 kHz upon NH_3_ exposure, suggesting weaker ammonia response capability. The corresponding sensing response is only 3% ± 0.1%, and begins to decay after day 8. This pronounced discrepancy is primarily attributed to the high susceptibility of pristine MXene to oxidation under air and NH_3_ exposure, which progressively reduces electrical conductivity and disrupts interfacial charge redistribution during gas adsorption. In contrast, the incorporation of In_2_O_3_ nanoparticles effectively alleviates oxidative degradation while facilitating interfacial charge transfer and increasing accessible active sites, thereby simultaneously enhancing response intensity and long‐term stability.

### 2.5 Analysis of NLoS Medium Interference Characteristics and Interference Self‐compensation

To evaluate the interference effects of different agricultural product packaging media on electromagnetic wave propagation, the RF transmission characteristics of various media were systematically investigated under 13.56 MHz. The underlying mechanisms of electromagnetic wave reflection and transmission were elucidated accordingly (Figure 5a). The reflection loss (*RL*) of the packaging media was calculated on the basis of transmission line theory [48, 49, 50]. Owing to the inherent differences in electromagnetic parameters among different packaging materials, particularly in relative permittivity (*ε_r_*), the terms *µ_r_/ε_r_* and *µ_r_ε_r_* in Equation (7) vary accordingly, leading to changes in the input impedance of electromagnetic waves within the media. As further indicated by Equation (8), the intrinsic mismatch between the input impedance of free space and that of agricultural packaging media (Figure S7) results in corresponding variations in *RL*. To further quantitatively assess the *RL* induced by different packaging media, a vector network analyzer (VNA) was employed to precisely measure the *S*‐parameters of the electromagnetic waves emitted by the near‐field probe, thereby enabling accurate extraction of the surface reflection loss [51].

(7) $$Z_{in}=Z_{o}\sqrt{\frac{\mu_{r}}{\varepsilon_{r}}}\tanh\left[j\left(\frac{2\pi }{C}\right)\sqrt{\mu_{r}\varepsilon_{r}}fd\right]$$

(8) $$RL\;\left(\mathrm{dB}\right)=-20\mathrm{lg}\left|S_{11}\left(\mathrm{dB}\right)\right|=20\mathrm{lg}\left|\frac{Z_{in}-Z_{o}}{Z_{in}+Z_{o}}\right|$$

where *Z_in_* is the input impedance, *µ_r_* is the relative permeability of the dielectric medium, *ε_r_* is the relative permittivity of the dielectric medium, *C* represents the transmission line structural parameters, *f* is the operating frequency, *d* is the transmission line length, and *Z_o_* is the characteristic impedance.

**FIGURE 5:**
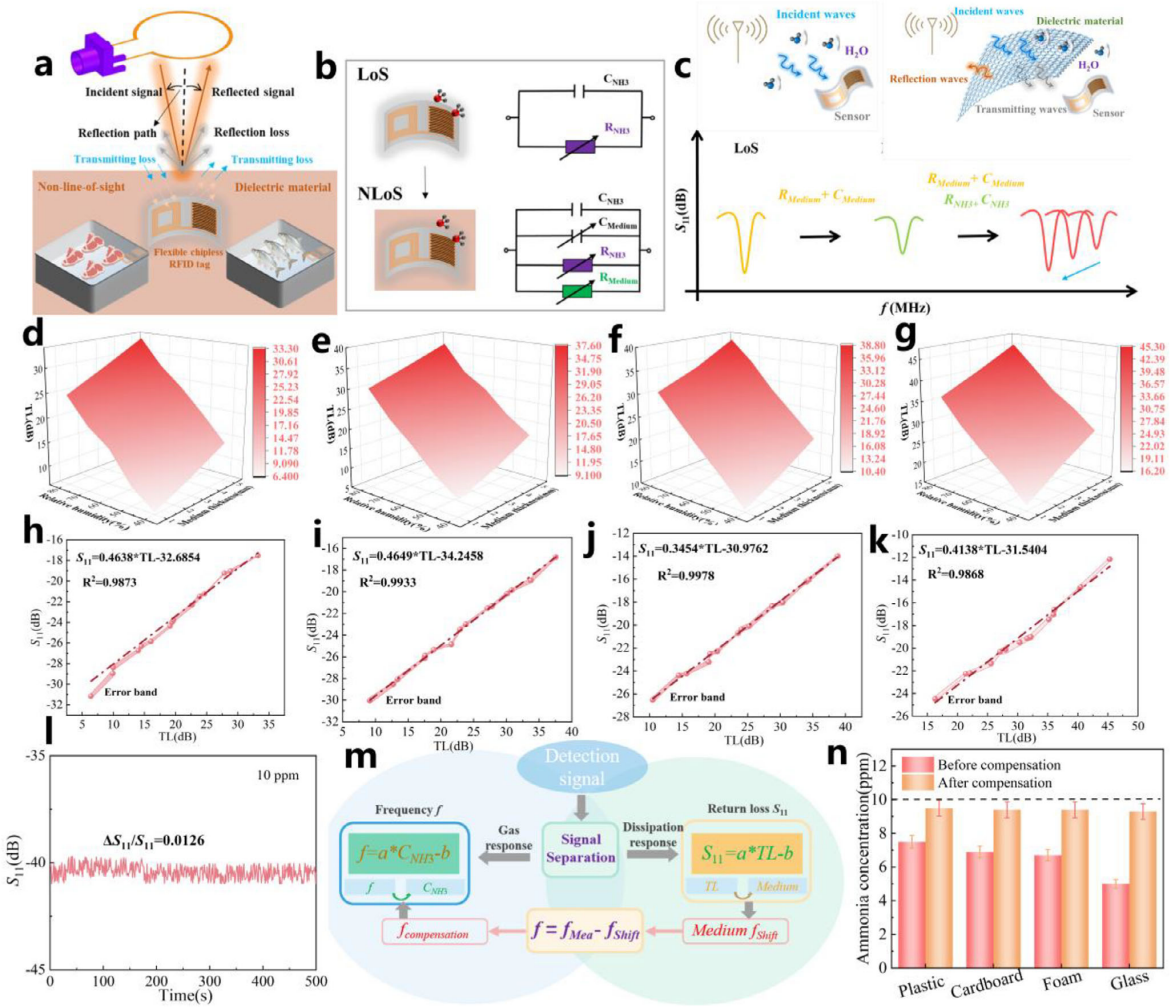
Schematic of electromagnetic wave propagation and the *S*_11_ amplitude–frequency signal self‐compensation mechanism under NLoS conditions: (a) Schematic of electromagnetic wave transmission through NLoS media, (b) Equivalent circuit models for LoS and NLoS passive detection, and (c) Frequency shift of RF signals. Electromagnetic wave transmission loss under different humidity and medium thickness conditions: (d) Plastic, (e) Cardboard, (f) Foam, and (g) Glass. Correlation mechanism between transmission losses of different media and the *S*_11_ amplitude response: (h) Plastic, (i) Cardboard, (j) Foam, and (k) Glass. (l) Ammonia adsorption induces variations in the amplitude of the *S*_11_ response under LoS conditions. (m) *S*_11_ amplitude‐frequency signal separation and self‐compensation schematic. (n) Comparison of ammonia detection concentration before and after compensation at 10 ppm.

According to electromagnetic wave propagation theory, electromagnetic waves experience energy dissipation when propagating through a medium due to the imaginary part of the complex permittivity (*εʺ*) of the material [52]. The propagation constant of electromagnetic waves in a medium can be expressed as *γ =* *α + jβ*, where the absorption behavior is governed by the attenuation constant *α* Equation (9). Variations in electromagnetic parameters, particularly the relative permittivity, among different packaging media affect the attenuation constant α, resulting in differences in absorption loss (*AL*), as described in the Equation (10).

(9) $$\alpha =\omega \sqrt{\mu \varepsilon^{\prime }}\sqrt{\frac{1}{2}\left(\sqrt{1+\tan\delta }-1\right)}$$

(10) $$AL\;\left(\mathrm{dB}\right)=k\alpha d$$

where *W =* 2*πf* and *µ = µ_o_*, where *µ* denotes the magnetic permeability and *µ_o_* is the magnetic permeability of free space (air). *k* represents the conversion coefficient, and *d* denotes the separation distance.

The NLoS medium interference is shown in Figure 5b. When electromagnetic waves encounter an NLoS medium during propagation, the pronounced mismatch in dielectric constants between the medium and air gives rise to partial reflection at the interface (Figure S8), whereas the transmitted waves undergo additional attenuation because of absorption losses as they propagate within the medium [53]. Moreover, under practical measurement conditions, increasing ambient humidity leads to the adsorption of water molecules on the surfaces of different packaging media, which in turn increases the effective dielectric constant and the dielectric loss tangent (tan δ) of the media (Figures S9, S10) [54]. This evolution significantly enhances electromagnetic wave propagation losses within the media, manifested as a simultaneous increase in reflection loss and absorption loss (Figures S11, S12). Specifically, owing to surface roughness and the presence of polar functional groups, food‐grade plastics (PETs) tend to form localized water films under high‐humidity conditions. In porous and hydrophilic materials such as food‐grade paperboard and expanded polystyrene (EPS), water molecules can readily accumulate within pore structures. Although food‐grade glass (borosilicate glass) has relatively high chemical and structural stability, the polarization of thin surface water films can still introduce additional absorption channels. As the thickness of the intervening medium increases, the electromagnetic wave propagation path length within the medium is extended, and the cumulative polarization effect of the water films leads to more pronounced energy attenuation. In terms of the equivalent circuit model, the introduction of an NLoS medium is equivalent to adding parallel resistance and capacitance to the LC resonator of the original LoS LC resonant circuit. Their combined effect manifests as a reduction in the effective resistance *R_Medium_* and an increase in the effective capacitance *C_Medium_*, thereby altering the resonance condition and impedance matching characteristics, which ultimately results in a shift in the sensor response (Figure 5c).

As shown in Figure 5d–g, the transmission loss of various packaging materials gradually increases with increasing ambient humidity and increasing material thickness. According to the underlying reflection and absorption loss mechanisms, medium‐induced electromagnetic interference primarily manifests as perturbations to the amplitude and resonance frequency of the RF signal. Notably, the dominant effect of medium‐induced interference loss is the variation in the RF signal amplitude. Furthermore, under LoS, the effect of ammonia adsorption on the *S*_11_ amplitude response was investigated (Figure 5l). The results demonstrate that NH_3_ adsorption induced an *S*_11_ amplitude variation of only 0.51 dB, corresponding to a relative change of approximately 0.0126 with respect to the baseline *S*_11_ level (−40.486 dB), thereby indicating a limited contribution to the overall amplitude response. This behavior is mainly attributed to the fact that NH_3_ adsorption primarily modulates the effective dielectric properties of the sensing structure, thereby mainly resulting in a shift of the resonant frequency. On this basis, an innovative *S*_11_ amplitude–frequency decoupling strategy is proposed to effectively separate medium‐induced interference signals from gas‐induced response signals. This approach enables the clear identification of frequency shifts induced by medium interference during NH_3_ sensing and provides a self‐compensating strategy for passive NH_3_ detection under NLoS interference conditions. Furthermore, a VNA was employed to analyze the attenuation behavior of the *S*_11_ amplitude signal under different medium interference conditions (Table SI). Subsequently, a correlation mechanism between the NLoS medium transmission loss and the *S*_11_ amplitude variation was established (Figure 5h–k). By exploiting the linear relationship between these two parameters and incorporating the transmission loss characteristics corresponding to different medium properties, variations in the *S*_11_ amplitude can be used to inversely infer medium attributes. Combined with the distinct frequency perturbation features induced by different media, this application enables effective self‐compensation of interference signals (Figures 5m and S13). To validate the feasibility of the proposed strategy, the performances of NH_3_ detection before and after self‐compensation were compared under various NLoS scenarios involving different medium properties at 25°C, 40% RH. The resulting concentration deviations are summarized in Figure 5n and demonstrate that after self‐compensation, the detection accuracy reaches the sub‐ppm level.

### 2.6 NLoS Detection of Fresh Agricultural Product Quality Deterioration

To verify the application feasibility of the flexible chipless RFID ammonia sensor in NLoS agricultural product packaging scenarios, as well as its ability to self‐compensate against NLoS medium interference, the sensing system was further extended to real biological and food matrices, with a particular emphasis on monitoring and evaluating the spoilage process of fresh meat (Figure 6a). TVB‐N is among the most important physicochemical indicators for assessing meat freshness [55, 56]. In this work, fresh fish fillets and beef slices were placed in Petri dishes and stored under ambient conditions for 16 h. Sensory evaluation and TVB‐N measurements were conducted at 4 h intervals, and the corresponding results are shown in Figure 6b,c, Note SIV. With increasing storage time, the degree of spoilage in fish and beef progressively intensified. During the early storage stage, the TVB‐N content increased slowly, followed by a rapid increase at later stages. Initially, the fish and the beef remained fresh, with very low TVB‐N levels. At 4 h, the TVB‐N content increased to 10.2 mg/100 g for fish and 9.7 mg/100 g for beef, while no obvious changes in color or odor were observed. At 8 h, the TVB‐N values reached 17.6 mg/100 g and 15.3 mg/100 g for fish and beef, respectively. Partial whitening appeared on the meat surfaces, accompanied by a slight off‐odor, indicating the onset of early spoilage. By 12 h, the TVB‐N content further increased to 25.9 mg/100 g for fish and 24.3 mg/100 × g for beef; both samples exhibited widespread surface whitening and pronounced fishy or putrid odors, suggesting a clearly spoiled state. At 16 h, the TVB‐N concentration sharply increased to 50.3 mg/100 g for fish and 47.6 mg/100 g for beef. Significant textural degradation was observed, with the samples becoming viscous and paste‐like, confirming severe spoilage (Figure 6d,e).

**FIGURE 6:**
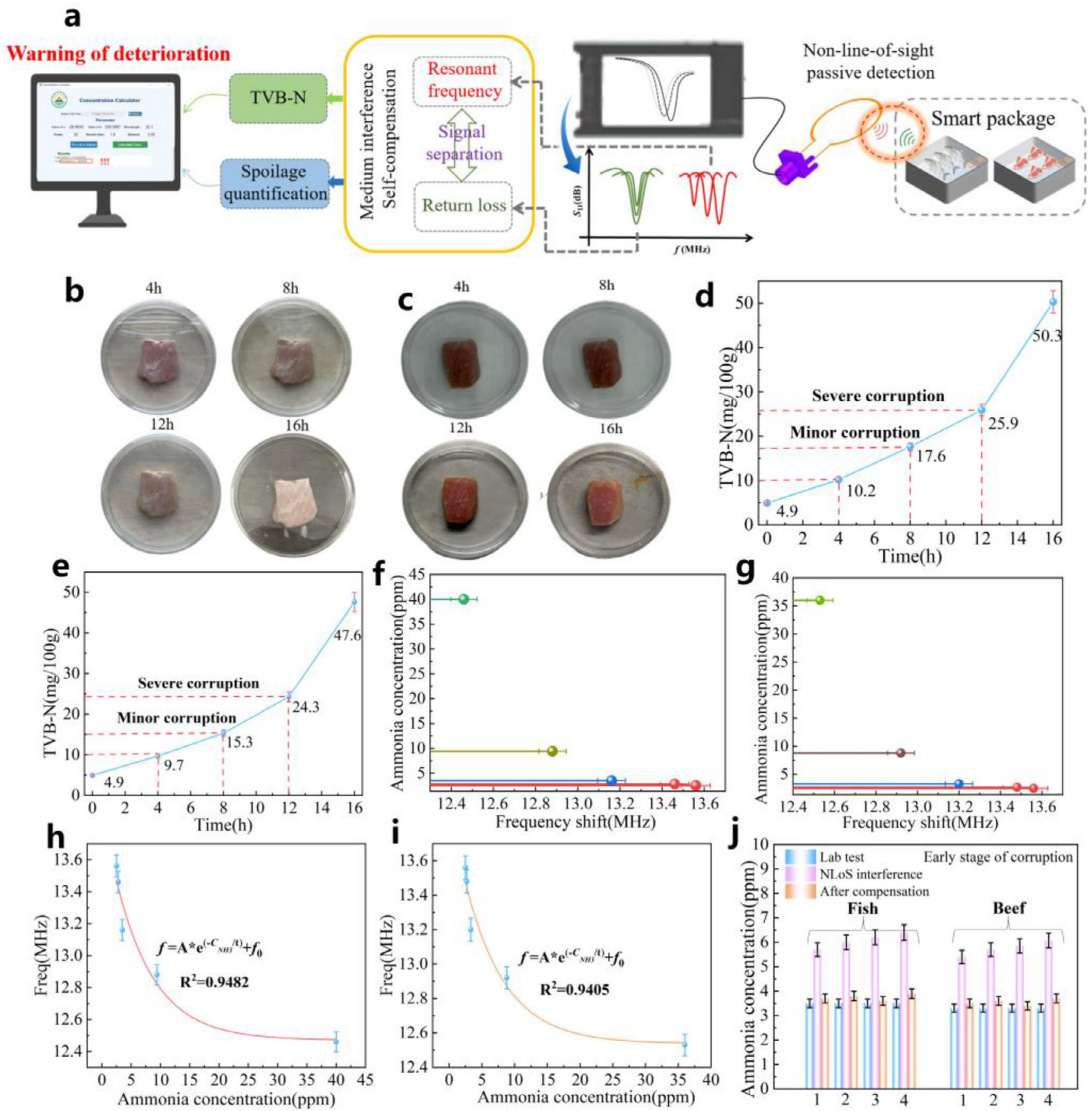
Analysis of NLoS ammonia detection and quality variation during fresh meat spoilage: (a) Schematic of NLoS passive detection of fresh produce quality deterioration, (b) Sensory evaluation of fish at different spoilage stages, (c) Sensory evaluation of beef at different spoilage stages, (d) Variation in TVB‐N concentration during fish spoilage, (e) Variation in TVB‐N concentration during beef spoilage, (f) Frequency shift variation with ammonia concentration during fish spoilage, (g) Frequency shift variation with ammonia concentration during beef spoilage, (h) Nonlinear ammonia concentration prediction model for fish based on frequency response, (i) Nonlinear ammonia concentration prediction model for beef based on frequency response, and (j) Ammonia detection compensation performance under NLoS conditions during the early spoilage stage of fish and beef. *Note*: in (j):1: Plastic, 2: Cardboard, 3: Foam, 4: Glass.

Combined with the results of volatile gas detection, the electromagnetic response evolution of the flexible chipless RFID ammonia sensor during the 0–16 h storage period was further analyzed. As shown in Figures S14a, S14b, the resonance frequency shift of the sensor was recorded at 4 h intervals. With the progressive spoilage of fish and beef, the resonance frequency gradually decreased over time. By correlating the TVB‐N values of fish and beef at each storage stage with the corresponding resonance frequency variations, a pronounced linear relationship was observed between the TVB‐N content and resonance frequency (Figures S15a, S15b). Moreover, an ammonia detector was used to monitor the NH_3_ concentration inside the sample chamber at 4 h intervals over the 0–16 h period. In parallel, a standard ammonia testing system was used to simulate the NH_3_ adsorption behavior of the sensor corresponding to each spoilage stage. As shown in Figure 6f,g, the resonance frequency similarly decreased with increasing ammonia concentration, and a highly linear correlation between the NH_3_ concentration and resonance frequency was obtained (Figures S15c, S15d). Based on the linear fitting results between TVB‐N concentration and *f*, as well as between NH_3_ concentration and *f*, a quantitative relationship between TVB‐N concentration and NH_3_ concentration can be established. Consequently, the TVB‐N concentration can be indirectly estimated by detecting the ammonia concentration, providing a reliable and nondestructive approach for assessing the freshness of fish and beef.

As illustrated in Figure S16, the flexible ammonia sensor was attached to the inner surface of food packaging to enable the passive detection of food quality deterioration. Under laboratory conditions, the dynamic variation in the ammonia concentration during meat spoilage was monitored. By correlating the frequency shift characteristics of the RF sensor under different ammonia concentrations (Figure 6f,g), a frequency‐response‐based nonlinear quantitative ammonia prediction model was established (Figure 6h,i). Error analysis of the proposed model was further conducted, as summarized in Tables SII and SIII, enabling the visual detection of ammonia concentration changes during fresh meat spoilage (Figure S17, Video S3). Further investigations were conducted to evaluate meat spoilage detection under NLoS packaging conditions. The experiments focused on the early stage of meat spoilage, during which ammonia release was predominant, and the dynamic RF signal shifts of the sensor were monitored across various packaging media. (Figures S18, S19). The RF signals were subsequently corrected using the previously proposed medium‐interference self‐compensation model. Thus, after self‐compensation, the detection accuracy for volatile ammonia released from fish and beef in different packaging media improved by 38.19% and 36.49%, respectively, thereby enabling passive NLoS detection of food quality deterioration. As shown in Figure 6j, after medium‐interference self‐compensation, the ammonia concentration measured by the sensor was in excellent agreement with the values obtained from laboratory reference instruments.

## 3 Conclusion

This work demonstrates a flexible chipless RFID ammonia sensor for NLoS sensing. By integrating an MXene/In_2_O_3_ composite sensing material with a flexible electromagnetic coupling architecture, passive detection of volatile ammonia is enabled even under packaging‐induced electromagnetic shielding conditions. A decoupled coupling configuration consisting of a spiral coil antenna and an LC interdigital electrode enables functional separation between electromagnetic energy transfer and gas sensing, thereby mitigating electromagnetic energy non‐uniformity and signal crosstalk associated with electromagnetic‐material response coupling. Concurrently, modulation of the electrical conductivity and interfacial impedance of the MXene/In_2_O_3_ composite amplifies ammonia adsorption–induced variations in equivalent electrical parameters, resulting in a 26.3% enhancement in sensor sensitivity. From an electromagnetic propagation perspective, the influence of packaging media on the *S*_11_ response under NLoS conditions is elucidated by distinguishing medium‐induced amplitude attenuation from resonant frequency shifts caused by ammonia adsorption. Based on this distinction, an *S*_11_ amplitude–frequency response separation method is proposed to decouple medium interference from gas sensing signals, enabling a self‐compensation strategy for passive ammonia detection under NLoS environments. Furthermore, a quantitative ammonia concentration prediction model is established using frequency response features and applied to freshness degradation scenarios of agricultural products, enabling visualization of ammonia evolution under NLoS conditions. This work enables passive, non‐destructive detection of quality deterioration in intelligent agricultural packaging under NLoS conditions, demonstrating potential for food safety monitoring.

## 4 Experimental Section

### 4.1 Sensor Fabrication

The passive sensor was fabricated on the basis of the screen‐printing process previously developed by our group [57]. The required equipment included a screen‐printing platform, a squeegee, copper‐based conductive ink, gold‐based conductive ink, a custom‐patterned screen mask, and a flexible polyimide (PI) substrate. First, the PI substrate was cut to an appropriate size based on the structural parameters of the tag derived from the HFSS simulations. The substrate was then immersed in deionized water and thoroughly cleaned using a magnetic stirrer to remove surface contaminants. Second, a predetermined amount of conductive copper ink was transferred into a separate container and uniformly stirred with a glass rod to improve dispersion and printing uniformity. The prepared ink and cleaned PI substrate were placed at designated positions on the screen‐printing platform. A squeegee was used to deposit the copper ink evenly on the PI surface following the predefined pattern. To prevent oxidation of the copper layer, a thin gold layer was subsequently printed onto the copper surface. Finally, the printed samples were subjected to thermal treatment in a constant‐temperature drying oven to facilitate the curing and solidification of the printed patterns, thereby ensuring the structural integrity of the flexible chipless RFID tag (Figure S20).

### 4.2 Materials Preparation

Aqueous ammonia solution (25–28 wt%) was purchased from Shanghai Macklin Biochemical Technology Co., Ltd. (China). Indium nitrate tetrahydrate (In(NO_3_)_3_·4H_2_O, 99.9% metal basis) was obtained from Shanghai Macklin Biochemical Technology Co., Ltd. (China). Ti_3_C_2_T_x_ MXene (purity: 99.9%) was obtained from Hefei Keliang New Materials Technology Co., Ltd. (China). VNA (R&S ZNLE14, Germany) with a sweep frequency range of 1–14 GHz was used for the RF measurements. Fresh freshwater fish and beef were purchased from a local supermarket in Hefei, China. The samples were first rinsed with deionized water and then sealed in plastic containers and stored in a refrigerator at 5°C for 2.0 h. This procedure was employed to induce rapid humane euthanasia of the fish while maintaining freshness. The fish and beef were subsequently sliced and placed in Petri dishes for subsequent experiments.

### 4.3 Material Synthesis

First, 0.8 g of In(NO_3_)_3_·4H_2_O was added to a mixed solvent comprising 30 mL of ethanol and 5 mL of 25 wt% NH_3_·xH_2_O. The mixture was continuously stirred at room temperature until a clear and transparent solution was obtained. This solution was subsequently transferred into a stainless‐steel autoclave lined with polytetrafluoroethylene and subjected to hydrothermal treatment at 100°C for 20 h. After naturally cooling to room temperature, the resulting solid product was collected and washed three times with deionized water and ethanol to remove residual impurities. The washed product was then dried in an oven at 80°C for 12 h to obtain an In(OH)_3_ precursor. The In(OH)_3_ precursor was subsequently calcined in a muffle furnace at 500°C for 2 h at a heating rate of 2°C min^−1^, yielding In_2_O_3_ nanoparticles (Figure S21a). Second, to prepare the MXene/In_2_O_3_ composite, Ti_3_C_2_T_x_ was mixed with a predetermined proportion of In_2_O_3_ nanoparticles and ground for 0.5 h to reactivate surface active sites. The resulting mixture was then dispersed in 15 mL of anhydrous ethanol and magnetically stirred for 5 h to promote uniform hybridization between Ti_3_C_2_T_x_ and In_2_O_3_ nanoparticles, resulting in the formation of a homogeneous Ti_3_C_2_T_x_/In_2_O_3_ hybrid suspension. Finally, the suspension was dried in a vacuum oven at 80°C for 12 h to obtain the MXene/In_2_O_3_ composite powder (Figure S21b).

### 4.4 Materials Characterization

The surface morphology and microstructure of the samples were examined using scanning electron microscopy (SEM, Hitachi SU8010, Japan) at an accelerating voltage of 5.0 kV. Transmission electron microscopy (TEM, FEI Tecnai G2 F20, USA) was employed at an accelerating voltage of 200 kV to further investigate the nanoscale morphology, crystal structure, and interfacial features. High‐resolution TEM (HRTEM) was used to obtain lattice fringes and detailed local structural information. Phase compositions were identified using an x‐ray diffractometer (XRD, Bruker D8 Advance, Germany) over a scanning range of 5°–90° in 2θ at a scan rate of 0.26°min^−1^, using Cu Kα radiation (*λ* = 0.15406 nm), with the instrument operated at 30 kV and 40 mA. The surface elemental composition and chemical states were analyzed by x‐ray photoelectron spectroscopy (XPS, Thermo ESCALAB 250XI, USA). In addition, Fourier transform infrared spectroscopy (FTIR, Thermo Scientific Nicolet 5700, USA) was used to characterize molecular structures and chemical bonding, while Raman spectroscopy (Renishaw inVia Reflex, UK) was performed to analyze molecular vibration modes and crystal structures.

### 4.5 Resistance Response Measurement During Ammonia Adsorption

To verify the resistance variation of the sensing material upon ammonia adsorption, an ammonia adsorption resistance measurement system was constructed, comprising a digital source meter (Siglent SMM3311X), a gas reaction chamber, a sensing tag, and a gas pump (Figure S5). Before the measurements, the digital source meter was mechanically calibrated with a standard calibration fixture. The interdigital electrodes of the sensing tag were electrically connected to the source meter. Nitrogen gas was then introduced into the reaction chamber to purge ambient air using a gas pump. Once the resistance signal stabilized, the baseline resistance was recorded. NH_3_ was subsequently introduced into the chamber, and the resistance change of the sensing tag loaded with MXene/In_2_O_3_ material upon ammonia adsorption was continuously monitored and recorded.

### 4.6 Determination of Dielectric Permittivity

To investigate variations in the dielectric permittivity of the packaging medium, the complex permittivity of the composite under different humidity conditions was characterized using the rectangular waveguide method. The sample was clamped in a dielectric coaxial fixture and connected to a VNA for measurement and analysis over a frequency range of 10 MHz to 14 GHz. (Figure S8).

### 4.7 Laboratory Ammonia Detection

Figure S3 illustrates the experimental platform for standard gas measurements. The testing system consisted of a dual‐channel gas mixing system, a reaction chamber, high‐pressure gas cylinders, a VNA, coaxial cables, a near‐field probe, a flexible chipless RFID ammonia sensor, and a computer for data acquisition and processing. In addition, a commercial ammonia gas detector (TGS2602‐B00**;** Figaro, Japan) was employed for reference measurements. Before the experiments, the VNA was calibrated using short, open, and load standards, the measurement frequency range was configured, and the near‐field probe and other components were properly connected. During the experiments, the gas mixing procedure was precisely controlled by high‐accuracy mass flow controllers to regulate the flow rates of the standard gases, ensuring thorough mixing of the background gas (N_2_) and the target gas within the measurement chamber. The air inside the reaction chamber was first completely purged, and then precalibrated mixed gas was introduced. After the flexible RF ammonia sensor was exposed to mixed gas and adsorption occurred, the *S*‐parameters of its spiral antenna were monitored in real time using a near‐field probe, while the corresponding response signals from the VNA were recorded simultaneously.

### 4.8 Calculation of Target Gas Concentration

Precise control of ppm‐level concentrations is achieved by adjusting the flow rate ratio between the two gas streams [58].

(11)

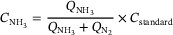

where Q_(NH3)_ and Q_(N2)_ represent the flow rates of ammonia and nitrogen, respectively, while C_(NH3)_ and C_(standard)_ denote the target ammonia concentration and the concentration of the standard gas.

### 4.9 NLoS Passive Ammonia Detection for Fresh Meat

As shown in Figure S17, block‐shaped fresh fish and beef samples were placed in storage containers made of different materials, with a flexible chipless RFID ammonia sensor attached to the inner wall of each container. Electromagnetic waves emitted by a near‐field probe penetrated the packaging medium and coupled with the spiral coil antenna, enabling real‐time acquisition of the tag's *S*‐parameters (data were collected after the RF signals reached a stable state). The measured data were transmitted from the VNA to a display terminal. Combined with the established quantitative ammonia concentration detection model, a gas concentration analysis program was developed, allowing visualization of the volatile ammonia concentration during the quality deterioration process of fresh meat.

## Author Contributions

G.H. conceived the idea and designed the overall research framework. G.H. and G.S. developed the methodology, carried out validation, and prepared the original draft of the manuscript. F.M. performed software implementation and data curation and contributed to manuscript revision. L.H. contributed to resource support and participated in manuscript revision. D.D. contributed to conceptual development, provided essential resources, and supervised the research activities. G.S., F.M., and D.D. contributed to methodology development, validation, and formal analysis, acquired funding, and critically revised the manuscript. All authors discussed the results and contributed to the final version of the manuscript.

## Conflicts of Interest

The authors declare no conflicts of interest.

## Supporting information




**Supplementary File**: advs76454‐sup‐0001‐SuppMat.docx.

**Supplementary File 2**: advs76454‐sup‐0002‐VideoS1.mp4.

**Supplementary File 3**: advs76454‐sup‐0003‐VideoS2.mp4.

**Supplementary File 4**: advs76454‐sup‐0004‐VideoS3.mp4.

## Data Availability

The data that support the findings of this study are available from the corresponding author upon reasonable request.

## References

[1] Y. Li , L. Guo , H. Yang , S. Chu , and X. Wang , “Multiscale Bioimpedance Detection Methods and Modeling for Dynamic Non‐destructive Monitoring of Agricultural Product Quality,” Trends in Food Science & Technology 157 (2025): 104888, 10.1016/j.tifs.2025.104888.

[2] Z. Wang , C. Jia , W. He , X. Zhang , and H. Feng , “Integrated Multi‐modal Flexible Sensors and AI‐driven Fusion Modeling for Internal and External Quality Detection of Agricultural Products,” Trends in Food Science & Technology 166 (2025): 105401, 10.1016/j.tifs.2025.105401.

[3] Z. He , J. Yu , X. Zhou , et al., “Multi‐sensor Fusion With Optimized Machine Learning for Non‐destructive Freshness Monitoring of Stored Korla Fragrant Pears,” Food Control 181 (2026): 111692, 10.1016/j.foodcont.2025.111692.

[4] Y. Hu and Z. Tan , “Enhanced Ammonia‐sensitive Intelligent Films Based on a Metal‐organic Framework for Accurate Shrimp Freshness Monitoring,” Food Chemistry 471 (2025): 142805, 10.1016/j.foodchem.2025.142805. 39798360

[5] G. Yang , L. Jiao , Y. Zhou , et al., “Non‐destructive Detection of Shrimp Freshness Based on Metal‐organic Framework Enrichment‐enhanced FTIR Spectroscopy,” Food Chemistry 485 (2025): 144426, 10.1016/j.foodchem.2025.144426. 40311578

[6] J. Xu , B. Mu , L. Zhang , R. Chai , Y. He , and X. Zhang , “Fabrication and Optimization of Passive Flexible Ammonia Sensor for Aquatic Supply Chain Monitoring Based on Adaptive Parameter Adjustment Artificial Neural Network (APA‐ANN),” Computers and Electronics in Agriculture 212 (2023): 108082, 10.1016/j.compag.2023.108082.

[7] G. Xuan , S. Ma , H. Lin , and J. Wang , “Identification of Potential Freshness Indicator of Atlantic Salmon Based on iTRAQ Proteomic Analysis,” European Food Research and Technology 249 (2023): 2661–2674, 10.1007/s00217-023-04320-y.

[8] Z. Iqbal , N. K. Afseth , A. Postelmans , et al., “Detection and Quantification of Pork Adulteration in Beef Meatballs With Raman Spectroscopy and near Infrared Spectroscopy,” Spectrochimica Acta Part A: Molecular and Biomolecular Spectroscopy 337 (2025): 126069, 10.1016/j.saa.2025.126069. 40154144

[9] X. Wang , L. Gong , and X. Zhou , “Ammonia Gas Sensor Fabricated by Multifunctional ZnO/GO Nanocomposites for Long‐Term, Self‐Powered Monitoring,” Advanced Science 13 (2025): 16833, 10.1002/advs.202516833. PMC12915130 41347596

[10] B. Paudel , S. Shen , Q. L. Tanjay , et al., “A Printable Chipless Sensor Label for Wireless Ammonia Detection and Seafood Monitoring,” Sensors and Actuators A: Physical 394 (2025): 116926, 10.1016/j.sna.2025.116926.

[11] R. Jia , W. Tian , H. Bai , J. Zhang , S. Wang , and J. Zhang , “Amine‐responsive Cellulose‐based Ratiometric Fluorescent Materials for Real‐time and Visual Detection of Shrimp and Crab Freshness,” Nature Communications 10 (2019): 795, 10.1038/s41467-019-08675-3. PMC6377604 30770837

[12] H. Zou , Z. Zhou , M. Huang , et al., “NFC/RFID‐enabled Wearables and Implants for Biomedical Applications,” Microsystems & Nanoengineering 11 (2025): 191, 10.1038/s41378-025-01010-5. PMC12549948 41130975

[13] E. Istif , H. Mirzajani , Ç. Dag , et al., “Miniaturized Wireless Sensor Enables Real‐time Monitoring of Food Spoilage,” Nature Food 4 (2023): 427–436, 10.1038/s43016-023-00750-9. 37202486

[14] W. Lv , J. Yang , X. Liu , et al., “Wireless Gas Sensors Enabled by Conjoined Conduction‐Polarization Mechanisms for Room Temperature Ppb‐Level Triethylamine Detection,” Nano Letters 25 (2025): 10025–10033, 10.1021/acs.nanolett.4c06676. 40498032

[15] W. Lv , J. Yang , Q. Xu , et al., “Wide‐range and High‐accuracy Wireless Sensor With Self‐humidity Compensation for Real‐time Ammonia Monitoring,” Nature Communications 15 (2024): 6936, 10.1038/s41467-024-51279-9. PMC11322651 39138176

[16] H. Nesser , H. A. Mahmoud , and G. Lubineau , “High‐Sensitivity RFID Sensor for Structural Health Monitoring,” Advanced Science 10 (2023): 2301807, 10.1002/advs.202301807. PMC10502838 37407517

[17] H. A. Mahmoud , H. Nesser , T. M. Mostafa , S. Ahmed , and G. Lubineau , “A Fully Printable Strain Sensor Enabling Highly‐Sensitive Wireless Near‐Field Interrogation,” Advanced Science 12 (2025): 2411346, 10.1002/advs.202411346. PMC11884597 39836642

[18] S. Meng , G. Ren , B. Hou , Y. Xue , Z. Song , and B. Chen , “A Sensitive Paper‐based Chipless RFID Sensor for Humidity Measurement at 2.45 GHz,” Sensors and Actuators B: Chemical 441 (2025): 138045, 10.1016/j.snb.2025.138045.

[19] W. Lv , Y. Zhang , H. Luo , et al., “Wide Remote‐Range and Accurate Wireless LC Temperature–Humidity Sensor Enabled by Efficient Mutual Interference Mitigation,” ACS Sensors 8 (2023): 4531–4541, 10.1021/acssensors.3c01200. 38006356

[20] W. Yue , Y. Guo , J.‐C. Lee , et al., “Advancements in Passive Wireless Sensing Systems in Monitoring Harsh Environment and Healthcare Applications,” Nano‐Micro Letters 17 (2025): 106, 10.1007/s40820-024-01599-8. PMC11712043 39779609

[21] Y. Ma , Z. Feng , B. Tian , et al., “LC Resonant Giant Magneto‐impedance Sensor With High SNR for Weak Magnetic Field Detection,” Measurement 262 (2026): 120105, 10.1016/j.measurement.2025.120105.

[22] X. Cheng , Y. Liu , W. Zhong , et al., “An Ultra‐Selective and Humidity‐Resistant Room‐Temperature‐Operated NO 2 Sensor Based on Black TiO 2,” Advanced Science 12 (2025): 09293, 10.1002/advs.202509293. PMC12591125 40787896

[23] H. Zhang , W. Cao , J. Wang , et al., “2D Conductive MOFs Intercalated in MXene Interlayer for Fast and Trace Detection of Triethylamine at Room Temperature,” Advanced Science 12 (2025): 2500786, 10.1002/advs.202500786. PMC12224948 40411411

[24] H. Lee , J. S. Lee , G. W. Kwak , et al., “Carbide‐Induced Thermal Shock Synthesis of High‐Entropy Alloy Nanoparticles Anchored on WO_3_ Nanofibers for High‐Performance Gas Sensors,” ACS Nano 12 (2025): 18095–18107, 10.1021/acsnano.4c11149. 40222014

[25] W. Y. Chen , X. Jiang , S.‐N. Lai , D. Peroulis , and L. Stanciu , “Nanohybrids of a MXene and Transition Metal Dichalcogenide for Selective Detection of Volatile Organic Compounds,” Nature Communications 11 (2020): 1302, 10.1038/s41467-020-15092-4. PMC7064528 32157089

[26] K. Khan , A. K. Tareen , W. Ahmad , et al., “Recent Advances in Non‐Ti MXenes: Synthesis, Properties, and Novel Applications,” Advanced Science 11 (2024): 2303998, 10.1002/advs.202303998. PMC11423233 38894594

[27] Y. Liu , K. Lee , H. Liu , et al., “Universal 3D‐Printing of Suspended Metal Oxide Nanowire Arrays on MEMS for AI‐Optimized Combinatorial Gas Fingerprinting,” Advanced Science 12 (2025): 11794, 10.1002/advs.202511794. PMC12631845 40859412

[28] Y. Seekaew , S. Kamlue , and C. Wongchoosuk , “Room‐Temperature Ammonia Gas Sensor Based on Ti_3_C_2_T_x_ MXene/Graphene Oxide/CuO/ZnO Nanocomposite,” ACS Applied Nano Materials 6 (2023): 9008–9020, 10.1021/acsanm.3c01637.

[29] Z. Yang , A. Liu , C. Wang , et al., “Improvement of Gas and Humidity Sensing Properties of Organ‐Like MXene by Alkaline Treatment,” ACS Sensors 4 (2019): 1261–1269, 10.1021/acssensors.9b00127. 30990023

[30] S. Kim , T. Y. Ko , A. K. Jena , et al., “Instant Self‐Assembly of Functionalized MXenes in Organic Solvents: General Fabrication to High‐Performance Chemical Gas Sensors,” Advanced Functional Materials 34 (2024): 2310641, 10.1002/adfm.202310641.

[31] W. Ren , J. Luan , L. Yin , et al., “Ultrasensitive Room‐Temperature NO 2 Gas Sensor Based on MXene–Cu 2 O Composites,” ACS Sensors 10 (2025): 3579–3588, 10.1021/acssensors.5c00215. 40249796

[32] J. Li , S. Li , L. Xie , et al., “A Wearable Fiber Sensor Employing MXene/in_2_O_3_ Composites for NH_3_ Detection in Kidney Disease Diagnosis,” Advanced Functional Materials 36 (2025): 24182, 10.1002/adfm.202524182.

[33] W. Huang , M. Yin , J. Xia , and X. Zhang , “A Review of Cross‐scale and Cross‐modal Intelligent Sensing and Detection Technology for Food Quality: Mechanism Analysis, Decoupling Strategy and Integrated Applications,” Trends in Food Science & Technology 151 (2024): 104646, 10.1016/j.tifs.2024.104646.

[34] G. Shi , X. Shen , L. Gu , S. Weng , and Y. He , “Multipath Interference Analysis for Low‐Power RFID‐Sensor Under Metal Medium Environment,” IEEE Sensors Journal 23 (2023): 20561–20569, 10.1109/JSEN.2023.3253571.

[35] M. Qin , L. Zhang , and H. Wu , “Dielectric Loss Mechanism in Electromagnetic Wave Absorbing Materials,” Advanced Science 9 (2022): 2105553, 10.1002/advs.202105553. PMC8981909 35128836

[36] F. Liu , Y. Li , S. Hao , et al., “Well‐aligned MXene/chitosan Films With Humidity Response for High‐performance Electromagnetic Interference Shielding,” Carbohydrate Polymers 243 (2020): 116467, 10.1016/j.carbpol.2020.116467. 32532396

[37] A. Cui , Y. Miao , C. Wang , et al., “NiFe_2_O_4_/Ni_3_Fe Nanoparticles Decorate Wood Carbon Strategies for Efficient Electromagnetic Absorption,” Chemical Engineering Journal 507 (2025): 160354, 10.1016/j.cej.2025.160354.

[38] J. Jiang , X. Deng , S. Li , X. Zeng , C. Wu , and C. Yang , “Hierarchically Porous Multiphase Si‐Based Ceramics With Synergistic Electromagnetic Wave Absorption Mechanisms,” Advanced Science 12 (2025): 10445, 10.1002/advs.202510445. PMC12622537 40810923

[39] X. Chen , L. Li , Y. Jiang , et al., “Manipulating the Local Electronic Structure Microenvironment at the MXene Interface to Achieve Efficient Anode for Vanadium Redox Flow Battery,” Journal of Energy Chemistry 104 (2025): 118–126, 10.1016/j.jechem.2024.11.062.

[40] C. Xue , W. Li , W. Sun , Y. Chen , W. Zheng , and Y. Jiang , “Chemisorbed Oxygen‐rich in_2_O_3_/MXene Co‐spinning Hetero‐nanofibers for Ultrafast Triethylamine Sensor,” Applied Surface Science 719 (2026): 165014, 10.1016/j.apsusc.2025.165014.

[41] R. Vishnuraj , K. K. Karuppanan , M. Aleem , and B. Pullithadathil , “Boosting the Performance of NO_2_ Gas Sensors Based on n–n type Mesoporous ZnO@In_2_O_3_ Heterojunction Nanowires: In Situ Conducting Probe Atomic Force Microscopic Elucidation of Room Temperature Local Electron Transport Gas Sensors Based on n–n Type Mesoporous ZnO@in_2_O_3_ Heterojunction Nanowires: In Situ Conducting Probe Atomic Force Microscopic Elucidation of Room Temperature Local Electron Transport,” Nanoscale Advances 2 (2020): 4785–4797, 10.1039/D0NA00318B. PMC9417526 36132937

[42] Z. Liu , T. He , H. Sun , B. Huang , and X. Li , “Layered MXene Heterostructured With In_2_O_3_ Nanoparticles for Ammonia Sensors at Room Temperature,” Sensors and Actuators B: Chemical 365 (2025): 131918, 10.1016/j.snb.2022.131918.

[43] M. Wu , M. He , Q. Hu , et al., “Ti3C2 MXene‐Based Sensors with High Selectivity for NH3 Detection at Room Temperature,” ACS Sensors 4 (2019): 2763–2770, 10.1021/acssensors.9b01308. 31564092

[44] L. Xu and X. Fu , “A Micro Resonant Gas Sensor With Adjustable Natural Frequency,” IEEE Transactions on Industrial Electronics 68 (2021): 5337–5345, 10.1109/TIE.2020.2988243.

[45] G. Hu , G. Shi , K. Li , D. Ding , W. Wu , and X. Tang , “Passive RFID Sensor Based on Loaded Short Circuit and Parasitic Unit for Ammonia Sensing Enhancement,” Sensors and Actuators A: Physical 382 (2025): 116167, 10.1016/j.sna.2024.116167.

[46] H. Zhang , Z. Zhang , Z. Li , H. Han , W. Song , and J. Yi , “A Chemiresistive‐potentiometric Multivariate Sensor for Discriminative Gas Detection,” Nature Communications 14 (2023): 3495, 10.1038/s41467-023-39213-x. PMC10264437 37311822

[47] X. Wei , M. Zhang , K. Chen , M. Huang , A. S. Mujumdar , and C. Yang , “Intelligent Detection and Control of Quality Deterioration of Fresh Aquatic Products in the Supply Chain: A Review,” Computers and Electronics in Agriculture 218 (2024): 108720, 10.1016/j.compag.2024.108720.

[48] C. Jia , F. Zhang , Z. Wang , et al., “Hollow Porous High‐entropy Metal Oxides Enhanced Synergistic Loss With Excellent Electromagnetic Wave Absorption,” Composites Communications 59 (2025): 102569, 10.1016/j.coco.2025.102569.

[49] Y. Guo , Y. Duan , X. Liu , et al., “Construction of Multiple Heterogeneous Interfaces Induced by rGO‐multimetallic LDH Derivatives to Improve Dielectric Loss for Enhanced Electromagnetic Wave Absorption,” Journal of Materials Science & Technology 237 (2025): 288–297, 10.1016/j.jmst.2025.01.086.

[50] W. Dong , S. Tu , S. U. Rehman , et al., “Balancing Electromagnetic Wave Loss Capability and Impedance Matching of MOF Derived Porous Carbon Composite FeSiB Based Soft Magnetic Alloys to Optimize the Absorption Performance,” Chemical Engineering Journal 517 (2025): 164370, 10.1016/j.cej.2025.164370.

[51] C. Zhang , W. Yang , B. Jiang , et al., “Structure‐driven Design of Cellular Graphene/Polystyrene Composites With Balanced Dielectric Loss and Impedance Matching for Enabling Multiband Electromagnetic Wave Absorption,” Chemical Engineering Journal 517 (2025): 164290, 10.1016/j.cej.2025.164290.

[52] Z. Yang , Z. Yang , N. Liu , G. Wang , K. Chen , and J. Liu , “Ultra‐broadband Metamaterial Absorber Utilizing ZrO_2_/Fe_3_O_4_ Composites and Resistive Films: Mechanistic Investigation of Electromagnetic Wave Dissipation,” Ceramics International 51 (2025): 59161–59169, 10.1016/j.ceramint.2025.10.134.

[53] Y. L. Li , J. C. Liu , D.‐S. Li , et al., “PDMS‐encapsulated Liquid Metal‐MXene Aerogels for Resilient Electromagnetic Wave Absorption in Harsh Environments,” Rare Metals 44 (2025): 3299–3312, 10.1007/s12598-024-03037-5.

[54] F. Bibi , C. Guillaume , N. Gontard , and B. Sorli , “Wheat Gluten, a Bio‐polymer to Monitor Carbon Dioxide in Food Packaging: Electric and Dielectric Characterization,” Sensors and Actuators B: Chemical 250 (2017): 76–84, 10.1016/j.snb.2017.03.164.

[55] Z. Wang , X. Wang , G. Qi , et al., “Nanoporous Graphene Oxide/Molybdenum Disulfide Based on Quartz Crystal Microbalance Gas Sensor for Total Volatile Basic Nitrogen Detection in Salmon Meat,” Journal of Food Composition and Analysis 142 (2025): 107417, 10.1016/j.jfca.2025.107417.

[56] J. Wang , T. Du , M. Sun , et al., “Europium/Beet Red Network as Dual‐mode Labels for Perishable Food Monitoring and Light‐induced Active Preservation,” Chemical Engineering Journal 521 (2025): 166918, 10.1016/j.cej.2025.166918.

[57] G. Shi , X. Shen , Y. He , and H. Ren , “Passive Wireless Detection for Ammonia Based on 2.4 GHz Square Carbon Nanotube‐Loaded Chipless RFID‐Inspired Tag,” IEEE Transactions on Instrumentation and Measurement 72 (2023): 1–12, 10.1109/TIM.2023.3300433.

[58] Z. Cheng , G. He , C. Liang , et al., “The WOA‐LSSVM Temperature Compensation Technology for Carbon Nanotube‐Based Ionizing Gas Sensor,” IEEE Sensors Journal 24 (2024): 35212–35220, 10.1109/JSEN.2024.3459940.

