# Supplementary material for: Non‐Line‐of‐Sight Passive Ammonia Sensor Loaded With MXene/In2O3 Composites for Agricultural Products Quality Deterioration Detection
Source: Adv Sci (Weinh). 2026 Jul 3:e76454. Online ahead of print. doi: 10.1002/advs.76454 (PMC13334600; doi:10.1002/advs.76454)
Supplement: Supplementary file 1 — Supplementary File: advs76454‐sup‐0001‐SuppMat.docx. [file ADVS-9999-e76454-s002.docx]

Supporting Information

**Non-Line-of-Sight Passive** **Ammonia Sensor Loaded with MXene/In_2_O_3_ Composites for** **Agricultural Products** **Quality Deterioration Detection**

*Guoping Hu ^a^, Lin He ^a^, Fanli Meng ^b *^, Daming Dong ^c *^, Guolong Shi ^a, d *^*

^a^ School of Artificial Intelligence, Anhui Agricultural University, Hefei, Anhui, 230036, China.

^b^ School of Information Science and Engineering, Northeastern University, Shenyang, 110819, China.

^c^ Intelligent Equipment Research Center, Beijing Academy of Agriculture and Forestry Sciences, Beijing, 100097, China.

^d^ Key Laboratory of Agricultural Sensors, Ministry of Agriculture and Rural, Hefei, 230036, China.

**Contents**

**Supplementary Figures:**

Supplementary **Figure S1.** Flexible chipless RFID sensor tag structure designed in HFSS

Supplementary **Figure S2.** Electromagnetic simulation of the interdigital electrode dimensions

Supplementary **Figure S3.** Ammonia passive detection in the laboratory

Supplementary **Figure S4.** Frequency shift of the chipless RFID sensor loaded with MXene/In_2_O_3_ and MXene

Supplementary **Figure S5.** Resistance response test of sensors to ammonia adsorption

Supplementary **Figure S6.** Signal strength test of flexible chipless RFID sensing tags under NLoS and LoS conditions

Supplementary **Figure S7.** Impedance analysis diagram of the packaging medium

Supplementary **Figure S8.** Rectangular waveguide dielectric constant testing system

Supplementary **Figure S9.** Real and imaginary parts of dielectric constants of various packaging media under different humidity conditions

Supplementary **Figure S10.** Tangent loss of different media under varying humidity

Supplementary **Figure S11.** Reflection loss of different media under varying humidity

Supplementary **Figure S12.** Absorption loss of different media under varying humidity and thickness

Supplementary **Figure S13.** Electromagnetic wave transmission loss variations under frequency perturbations for different media

Supplementary **Figure S14.** Resonant frequency variations of meat spoilage with TVB-N content

Supplementary **Figure S15.** Linear correlation between frequency and spoilage indicators

Supplementary **Figure S16.** NLoS passive ammonia detection of fresh meat

Supplementary **Figure S17.** Visualization and analysis of fresh meat quality assessment

Supplementary **Figure S18.** Dynamic variations of RF sensor signals reflecting fresh fish quality deterioration under different packaging media

Supplementary **Figure S19.** Dynamic variations of RF sensor signals reflecting fresh beef quality deterioration under different packaging media

Supplementary **Figure S20.** Fabrication of flexible chipless RFID sensor tags

Supplementary **Figure S21.** Preparation of MXene/In_2_O_3_ composite material

Supplementary **Figure S22.** Mechanism of humidity-enhanced sensing response

Supplementary **Figure S23.** Mechanism of temperature fluctuation-induced sensing response

**Supplementary Notes:**

Supplementary **Note I.** Percentage calculation of gas response of the sensor

Supplementary **Note II.** Ammonia adsorption process of MXene/In_2_O_3_ materials under high-humidity conditions

Supplementary **Note III.** Temperature fluctuation-induced ammonia adsorption process in MXene/In_2_O_3_ materials

Supplementary **Note IV.** **Determination of TVB-N content in fresh fish and beef**

**Supplementary Tables:**

Supplementary **Table I.** Analysis of *S*_11_ amplitude corresponding to medium transmission loss

Supplementary **Table II.** Error analysis of a nonlinear ammonia quantification model based on frequency responses associated with fish freshness deterioration

Supplementary **Table III.** Error analysis of a nonlinear ammonia quantification model based on frequency responses associated with beef freshness deterioration


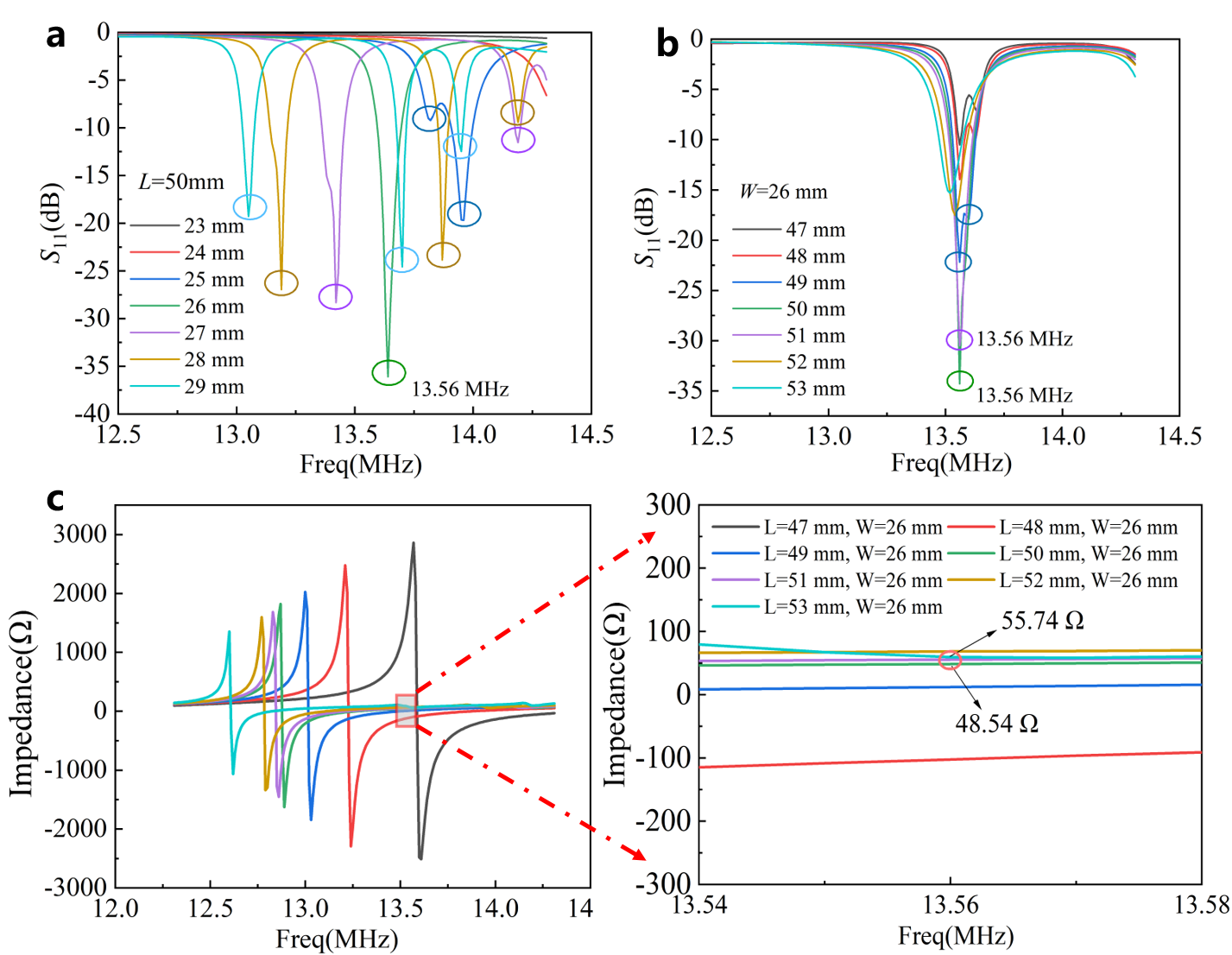


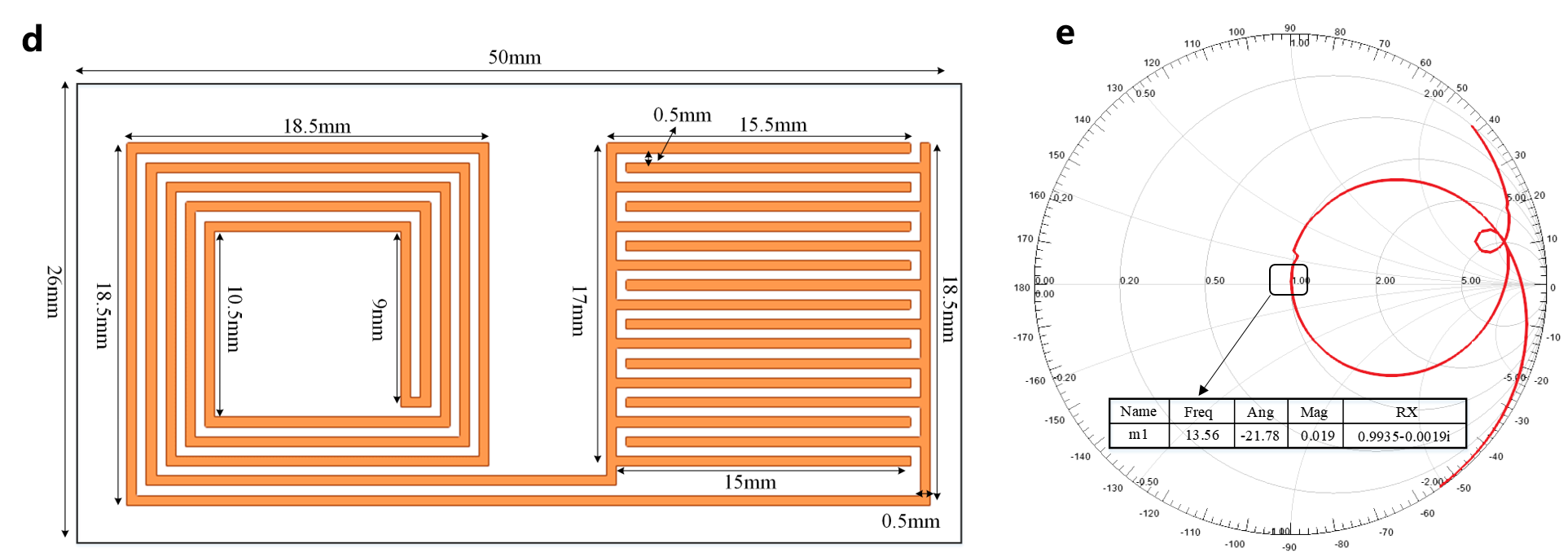


**Figure S1.** Flexible chipless RFID sensor tag structure designed in HFSS: a) and b) Dimension design, c) Impedance characteristics analysis, d) Geometrical structure of the flexible chipless RFID sensor tag, e) Smith chart of impedance


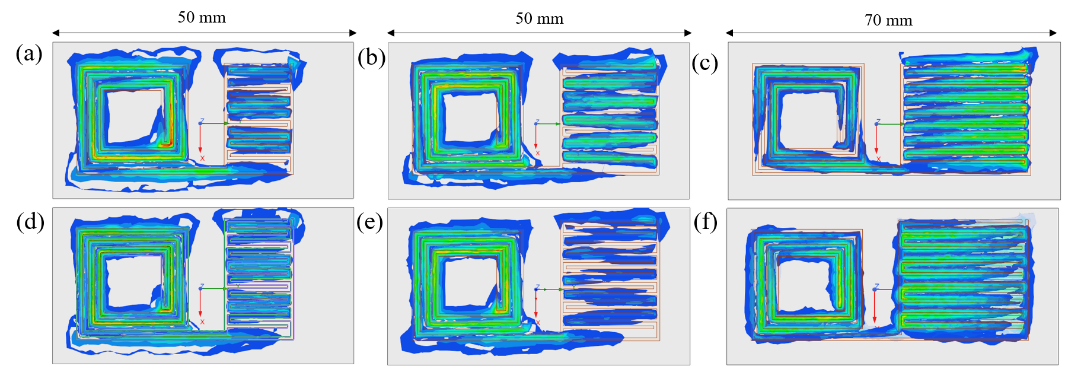


**Figure S2.** Electromagnetic simulation of the interdigital electrode dimensions: a) *L*=10 mm, *g*=0.5 mm, b) *L*=15 mm, *g*=0.5 mm, c) *L*=20 mm, *g*=0.5 mm, d) *L*=10 mm, *g*=0.6 mm, e) *L*=15 mm, *g*=0.6 mm, f) *L*=20 mm, *g*=0.6 mm


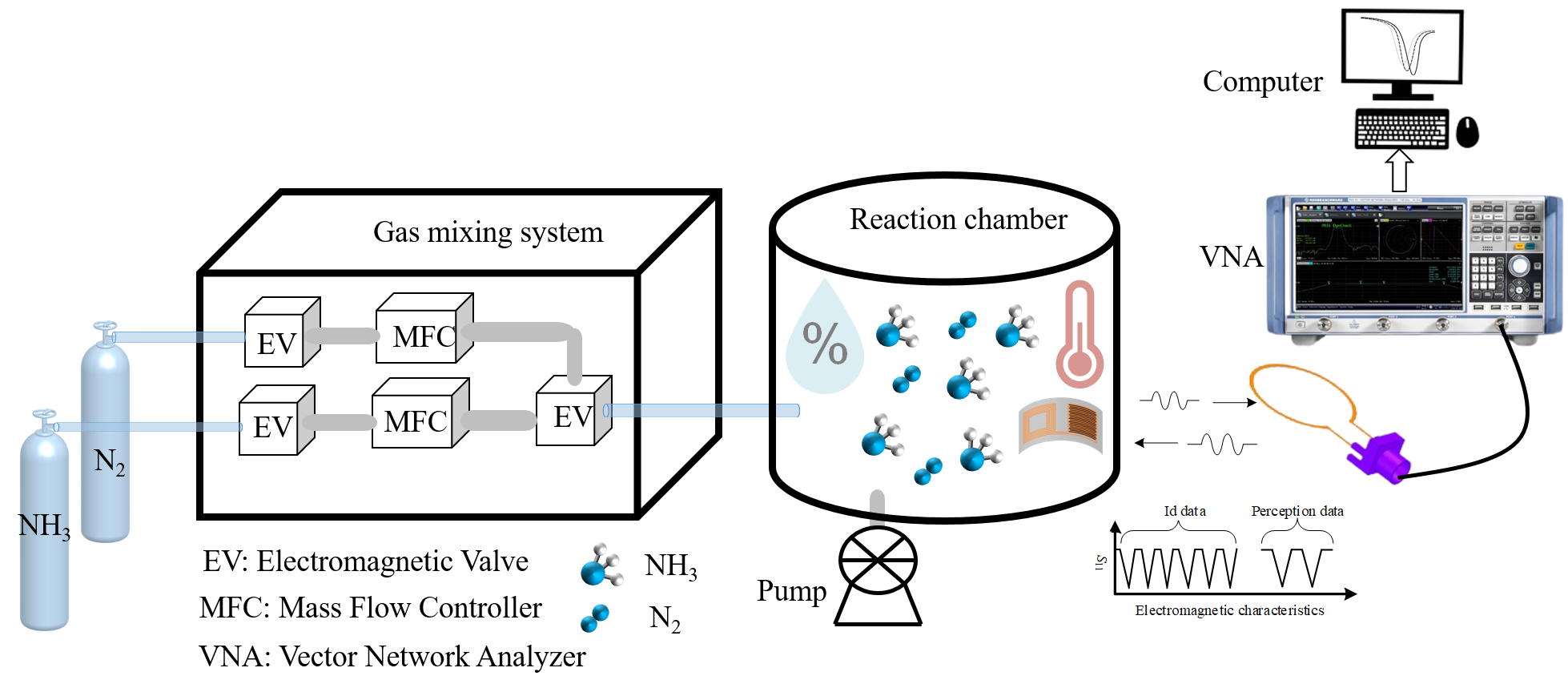


**Figure S3.** Ammonia passive detection in the laboratory


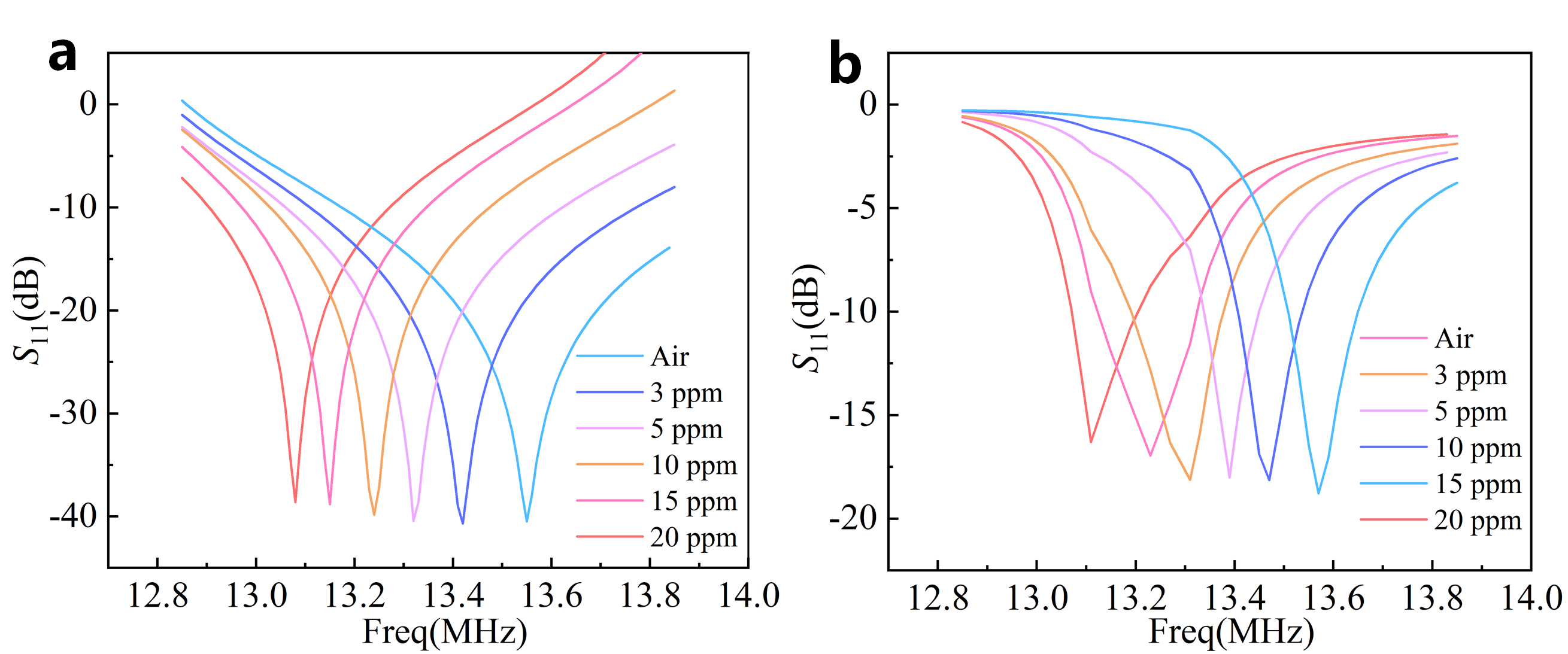


**Figure S4.** a) Frequency shift of the chipless RFID sensor loaded with MXene/In_2_O_3_ composite material, b) Frequency shift of the chipless RFID sensor loaded with MXene material


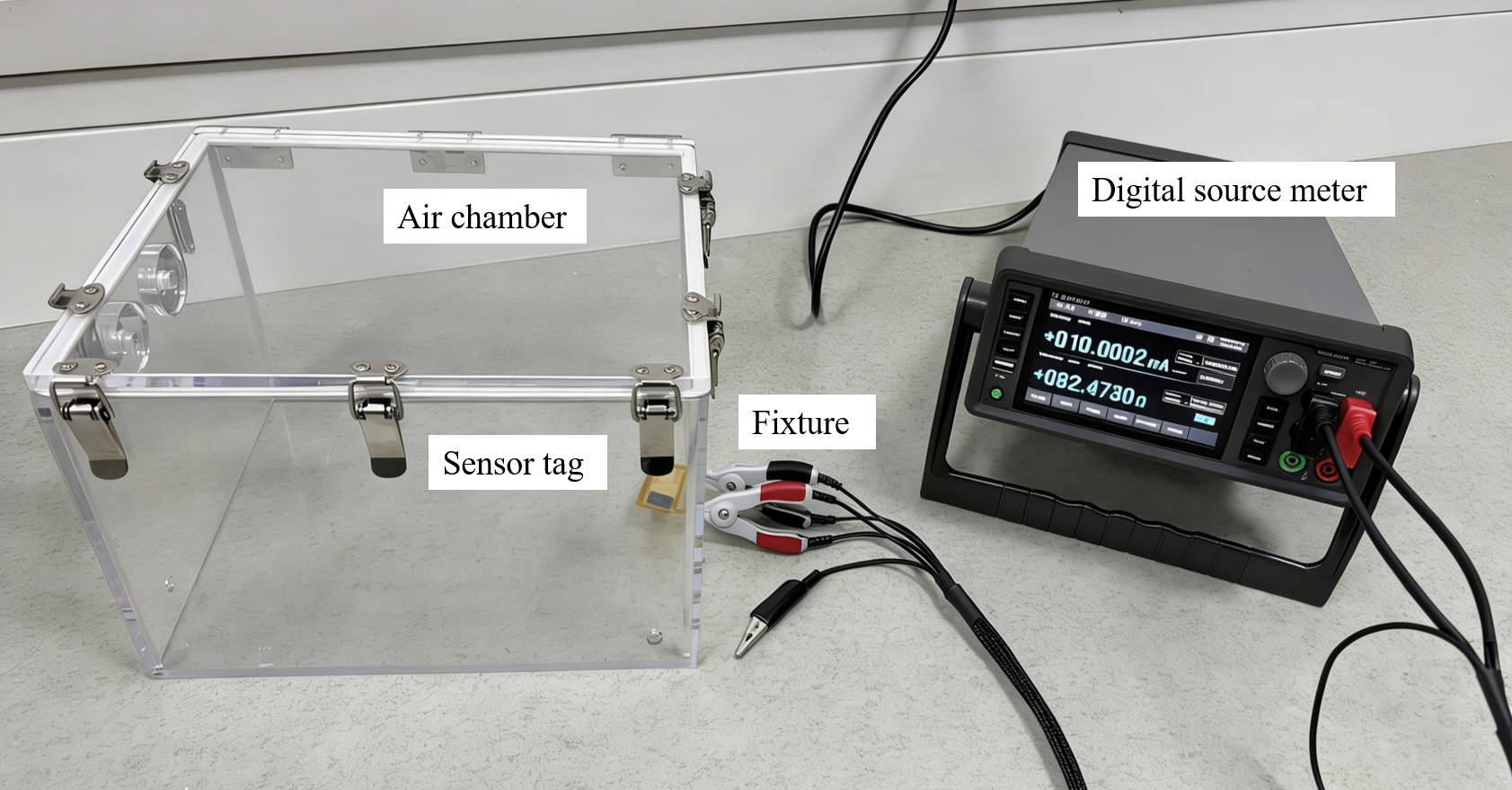


**Figure S5.** Resistance response test of sensors to ammonia adsorption


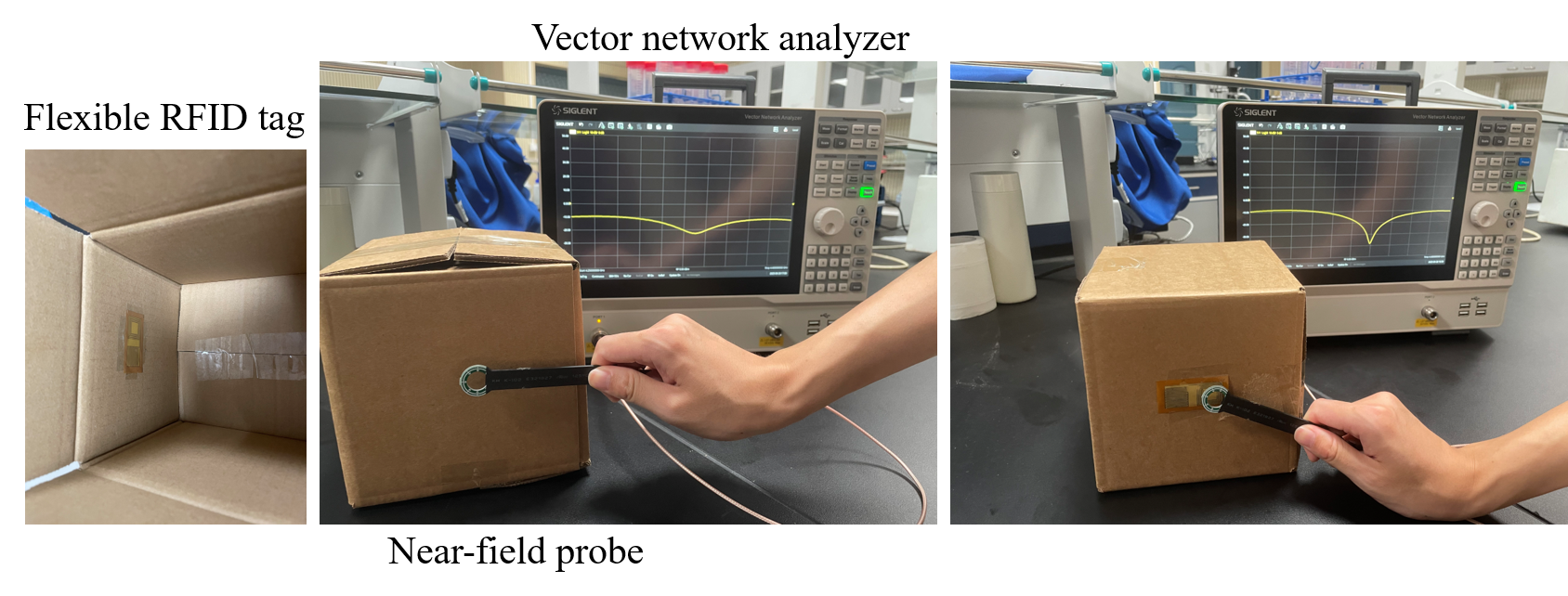


**Figure S6.** Signal strength test of flexible chipless RFID sensing tags under NLoS and LoS conditions


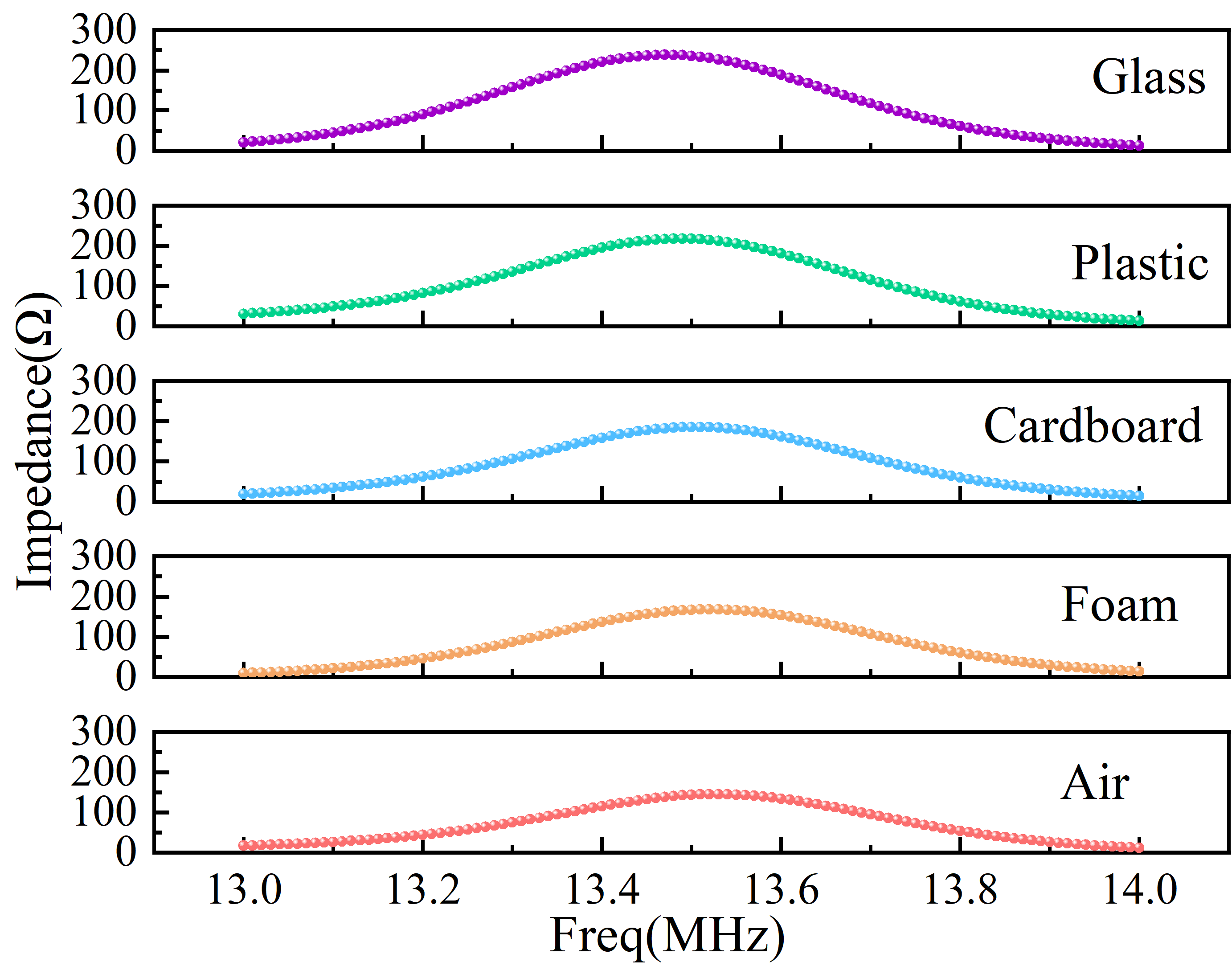


**Figure S7.** Impedance analysis diagram of the packaging medium


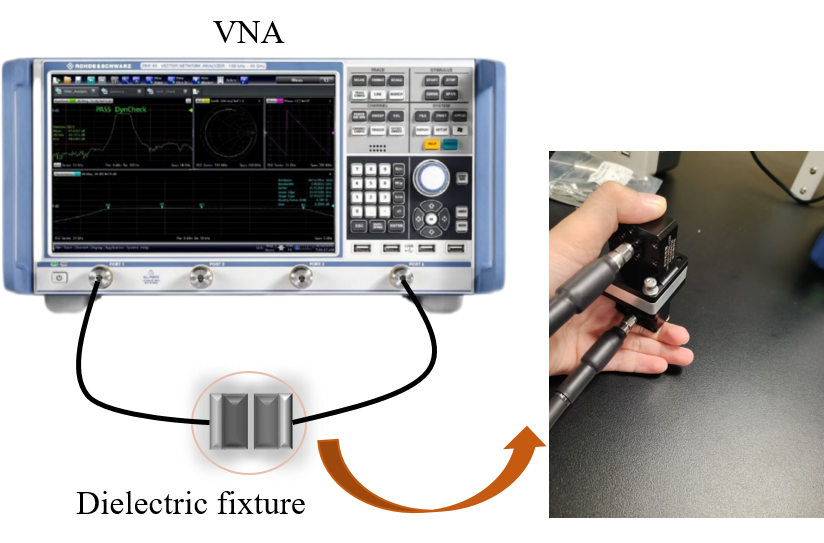


**Figure S8.** Rectangular waveguide dielectric constant testing system


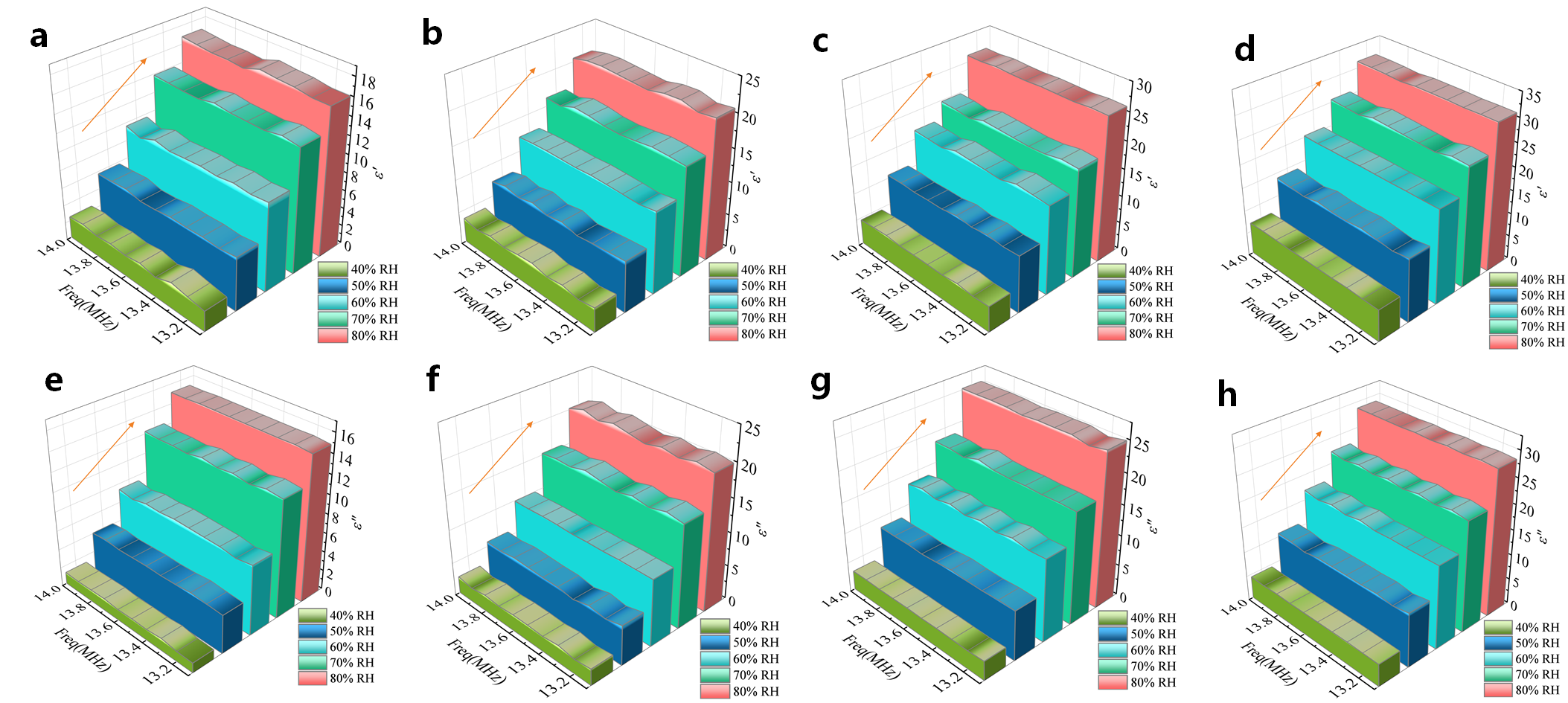


**Figure S9.** Real part of dielectric constants of various packaging media under different humidity conditions: a) Plastic, b) Cardboard, c) Foam, d) Glass, Imaginary part of dielectric constants of various packaging media under different humidity conditions: e) Plastic, f) Cardboard, g) Foam, h) Glass


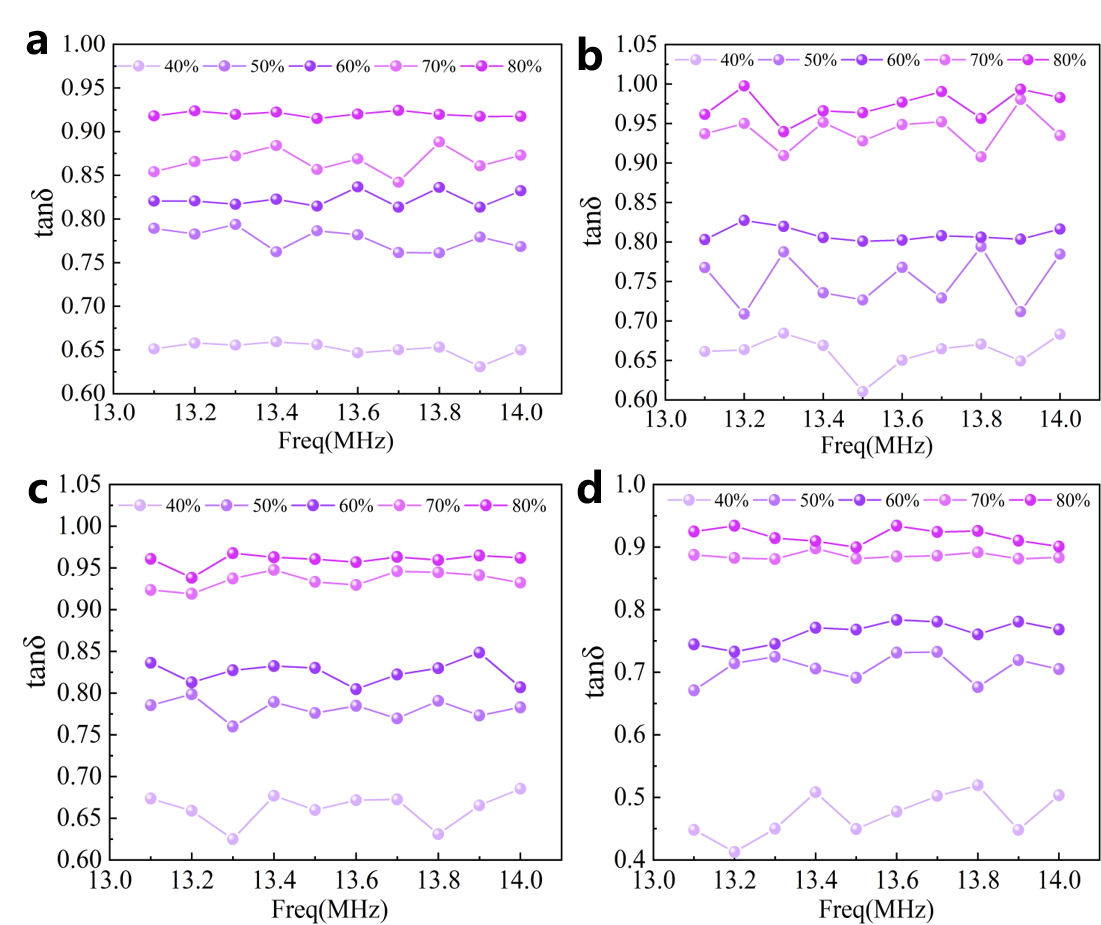


**Figure S10.** Tangent loss of different media under varying humidity: a) Plastic, b) Cardboard, c) Foam, d) Glass


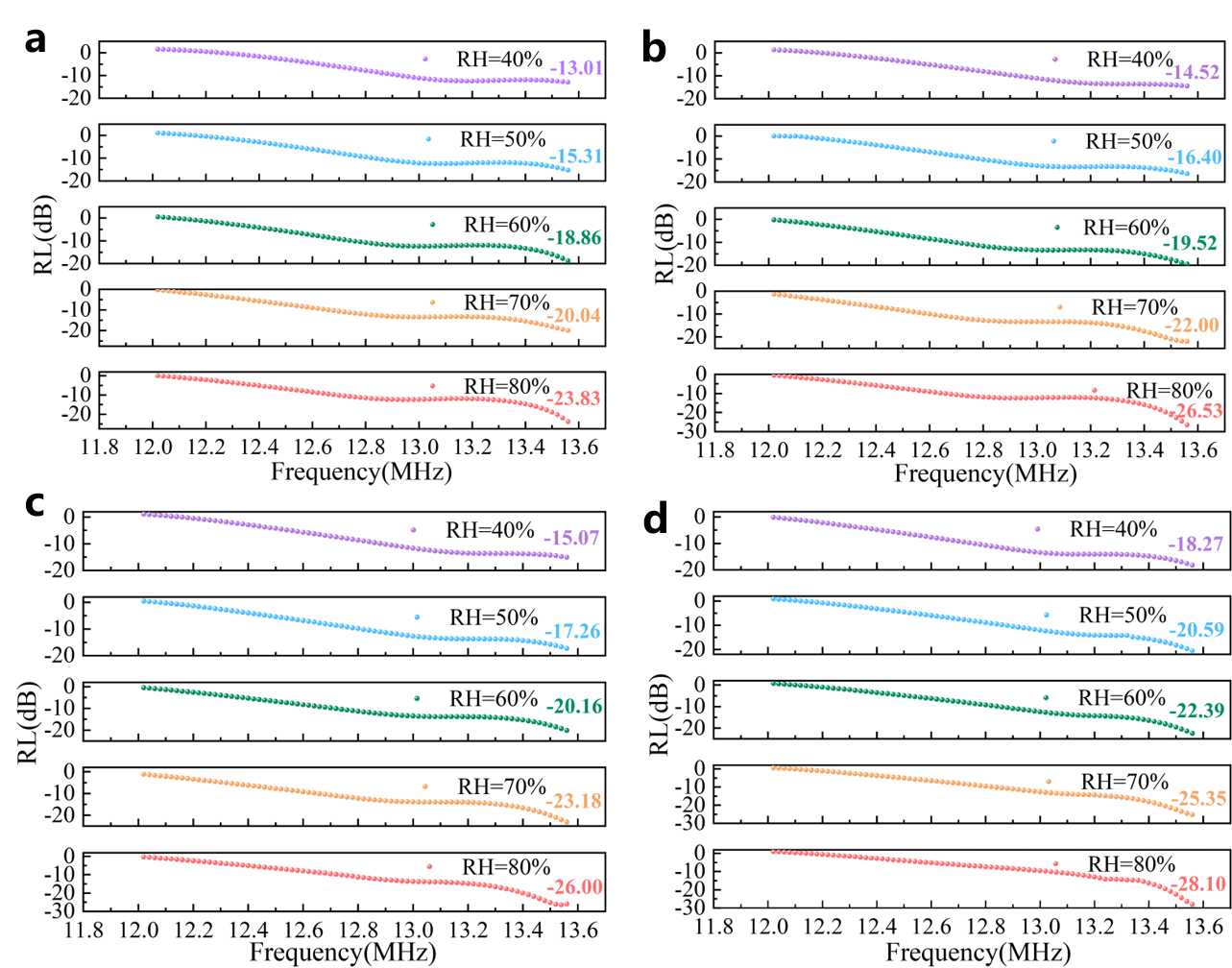


**Figure S11.** Reflection loss of different media under varying humidity: a) Plastic, b) Cardboard, c) Foam, d) Glass


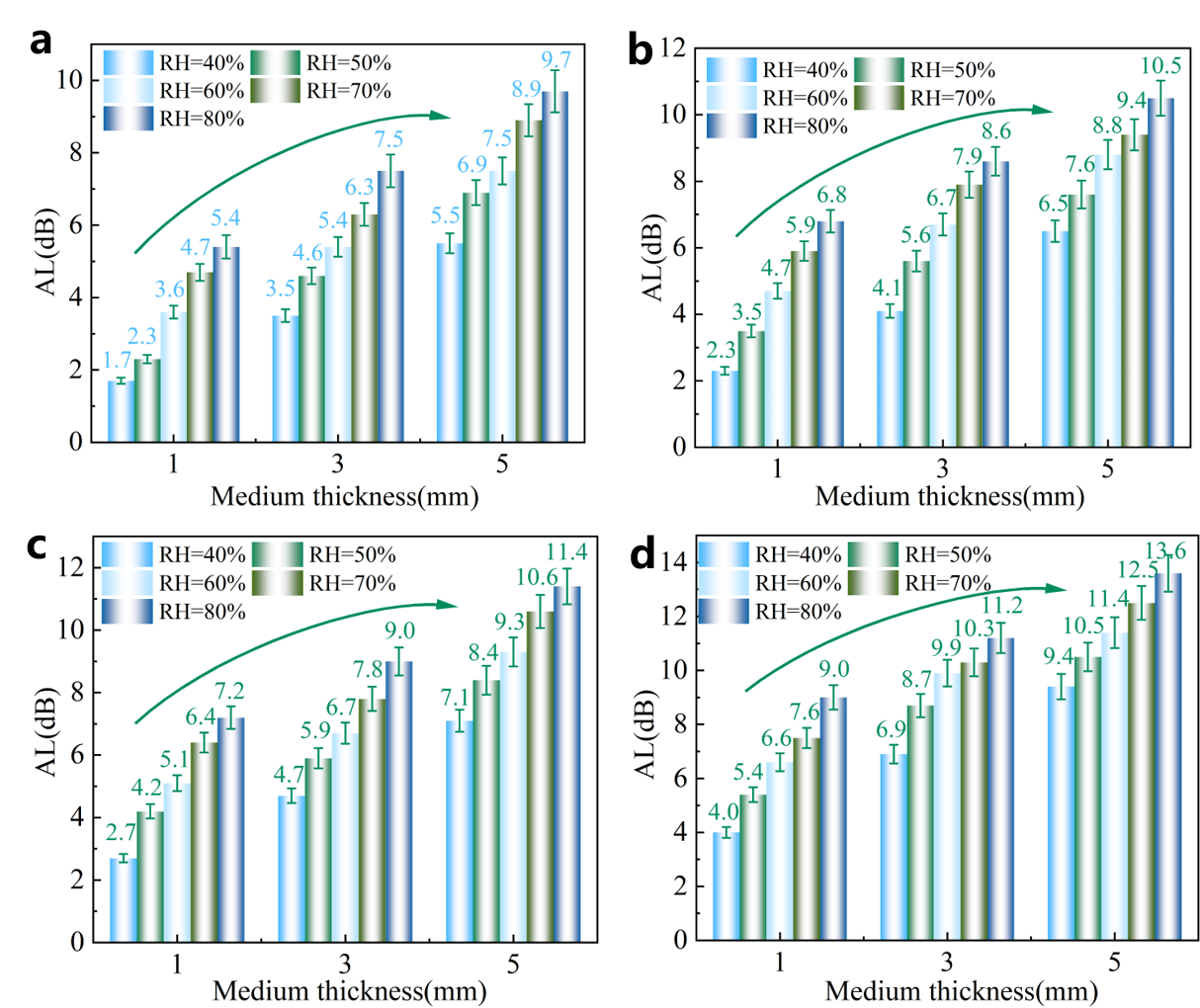


**Figure S12.** Absorption loss of different media under varying humidity and thickness: a) Plastic, b) Cardboard, c) Foam, d) Glass


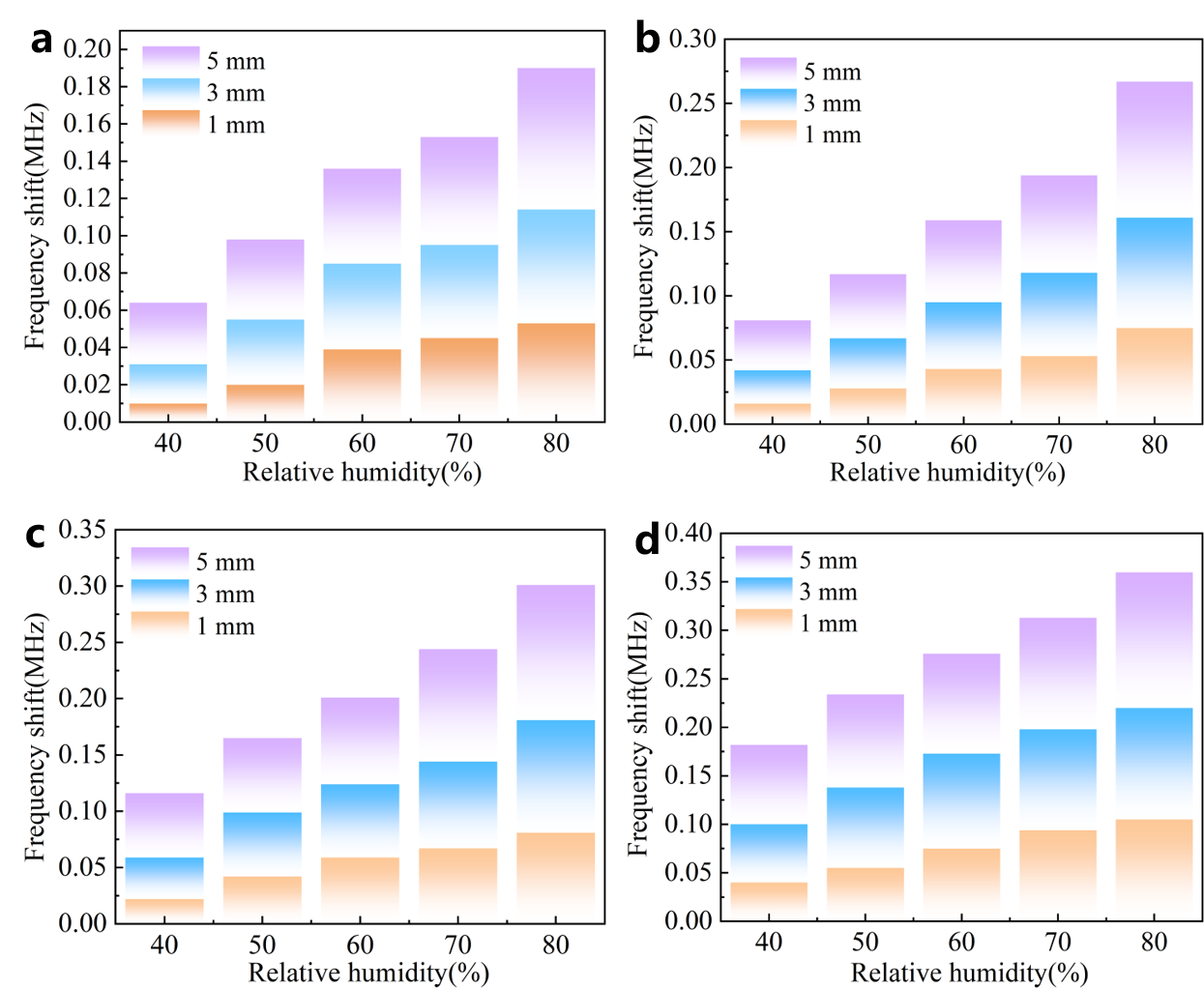


**Figure S13.** Electromagnetic wave transmission loss variations under frequency perturbations for different media: a) Plastic, b) Cardboard, c) Foam, d) Glass


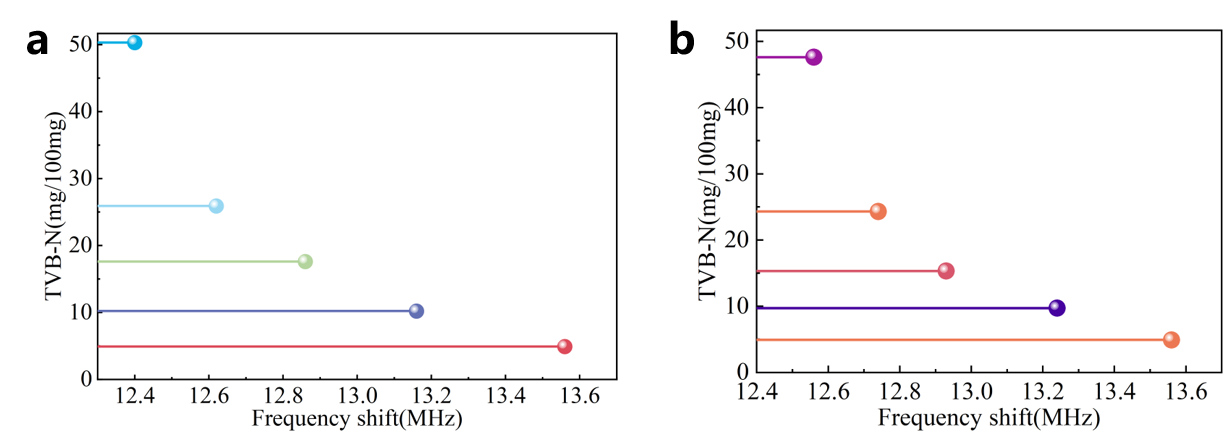


**Figure S14.** Resonant frequency variations of meat spoilage with TVB-N content: a) Fish, b) Beef


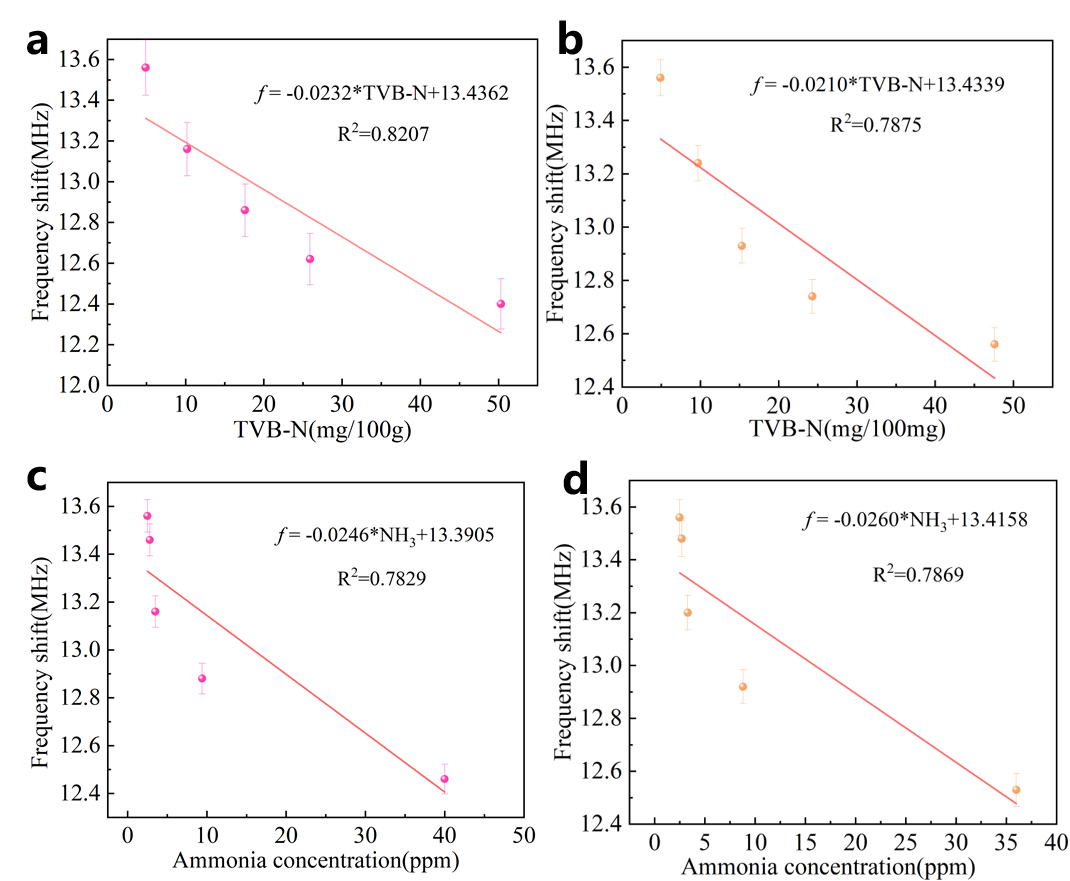


**Figure S15.** Linear correlation between frequency and spoilage indicators: a) TVB-N in fish, b) TVB-N in beef, c) Ammonia content in fish, d) Ammonia content in beef


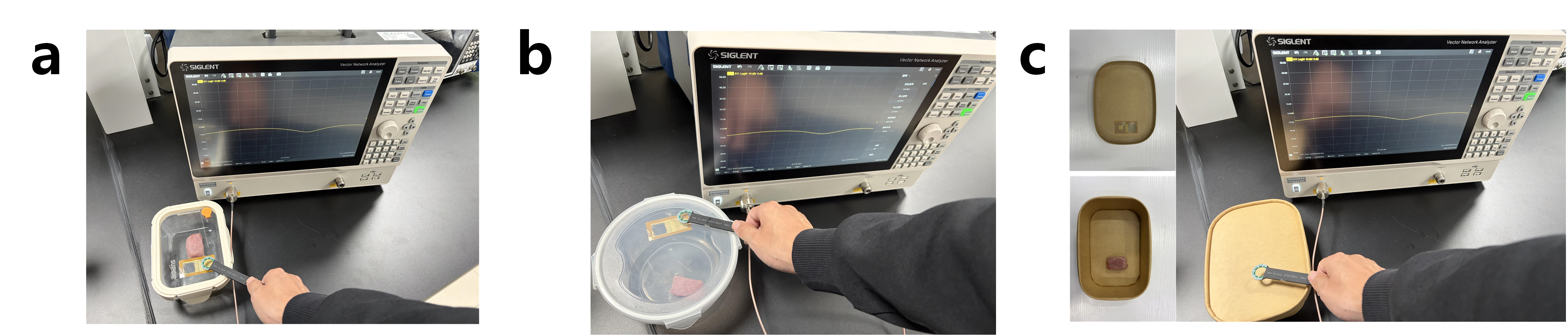


**Figure S16.** NLoS passive ammonia detection of fresh meat


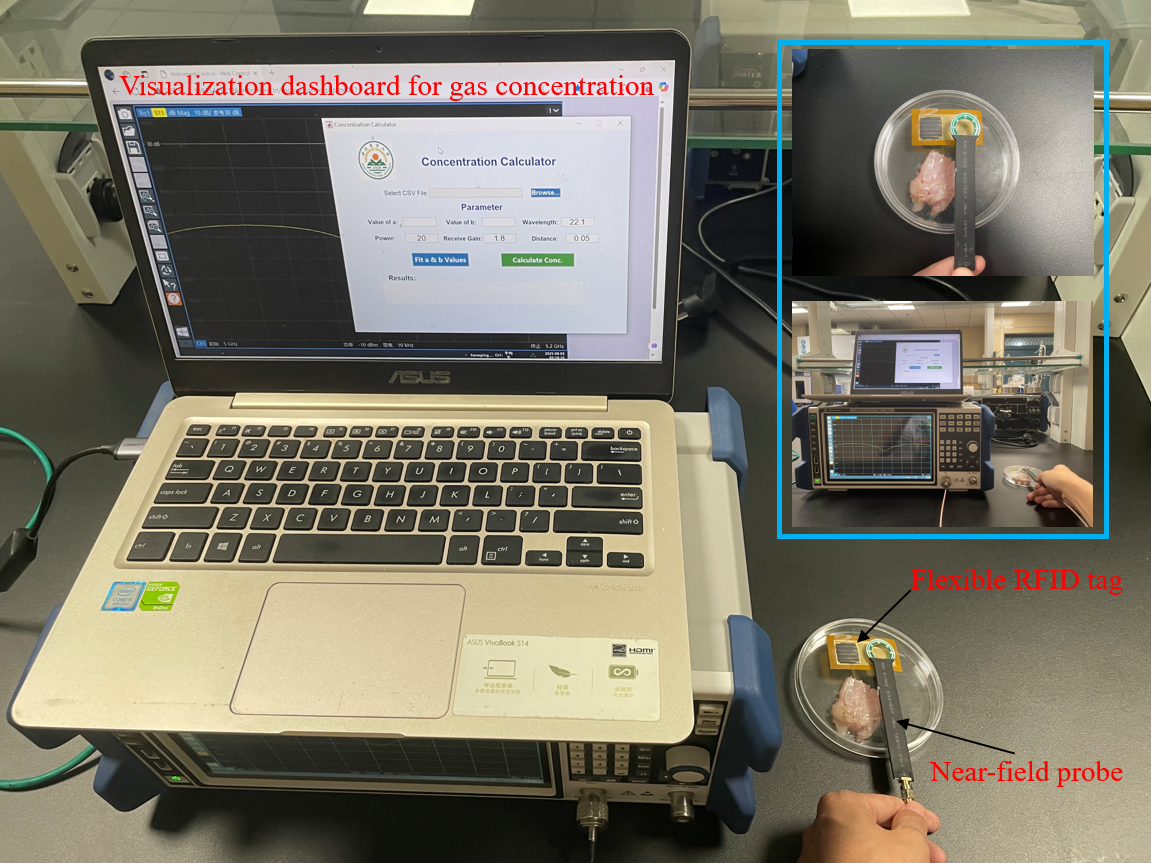


**Figure S17.** Visualization and analysis of fresh meat quality assessment


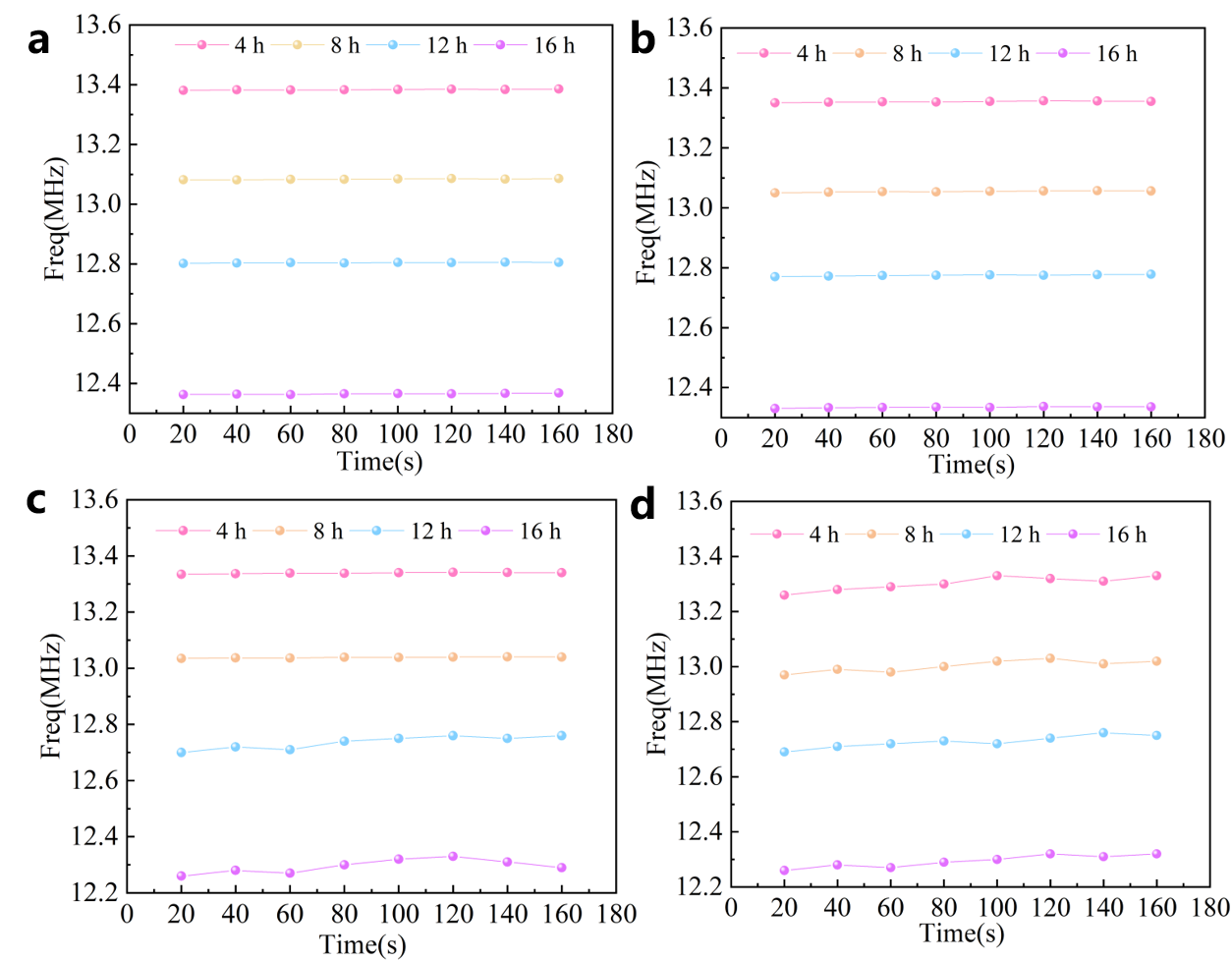


**Figure S18.** Dynamic variations of RF sensor signals reflecting fresh fish quality deterioration under different packaging media: a) Plastic, b) Cardboard, c) Foam, d) Glass


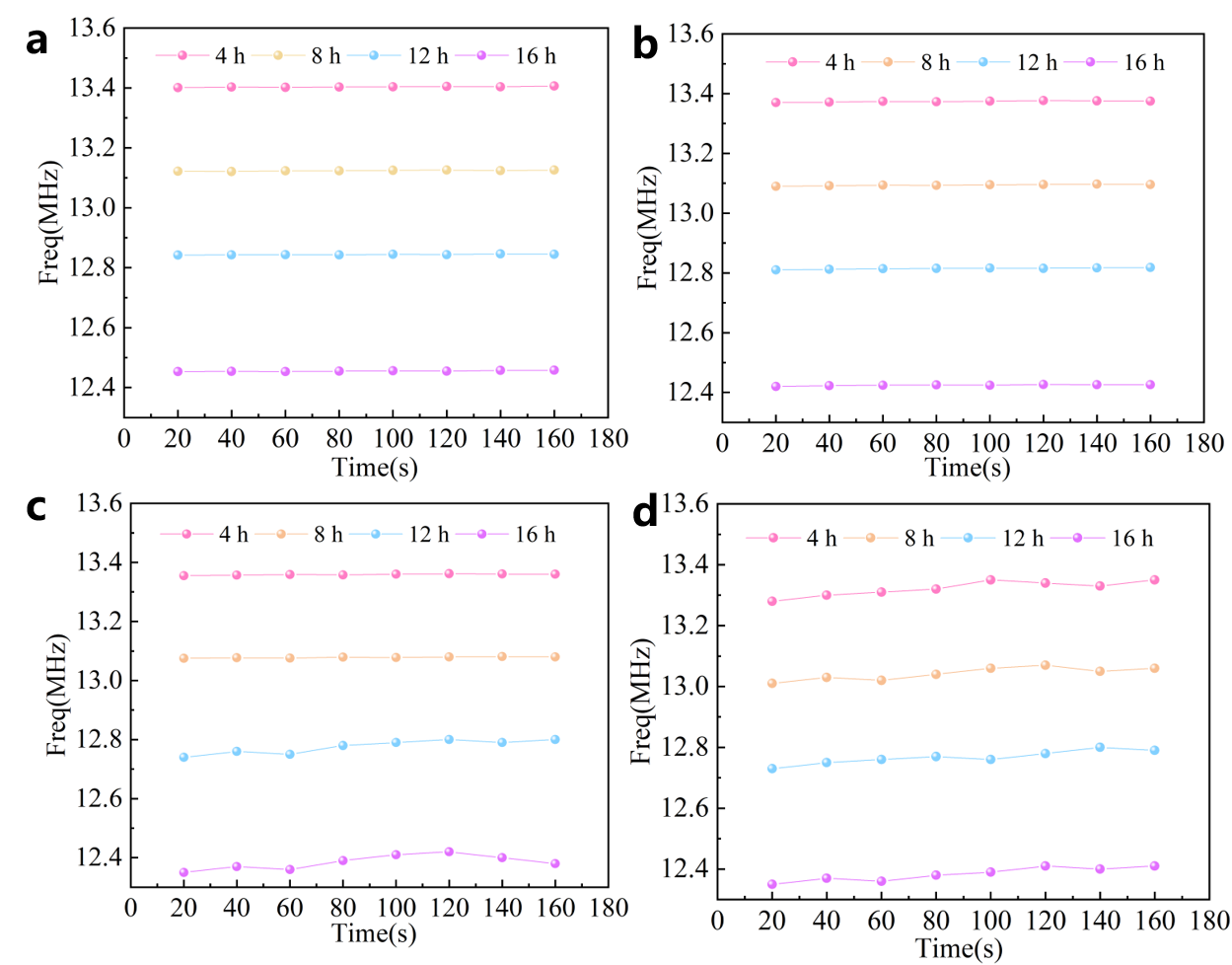


**Figure S19.** Dynamic variations of RF sensor signals reflecting fresh beef quality deterioration under different packaging media: a) Plastic, b) Cardboard, c) Foam, d) Glass


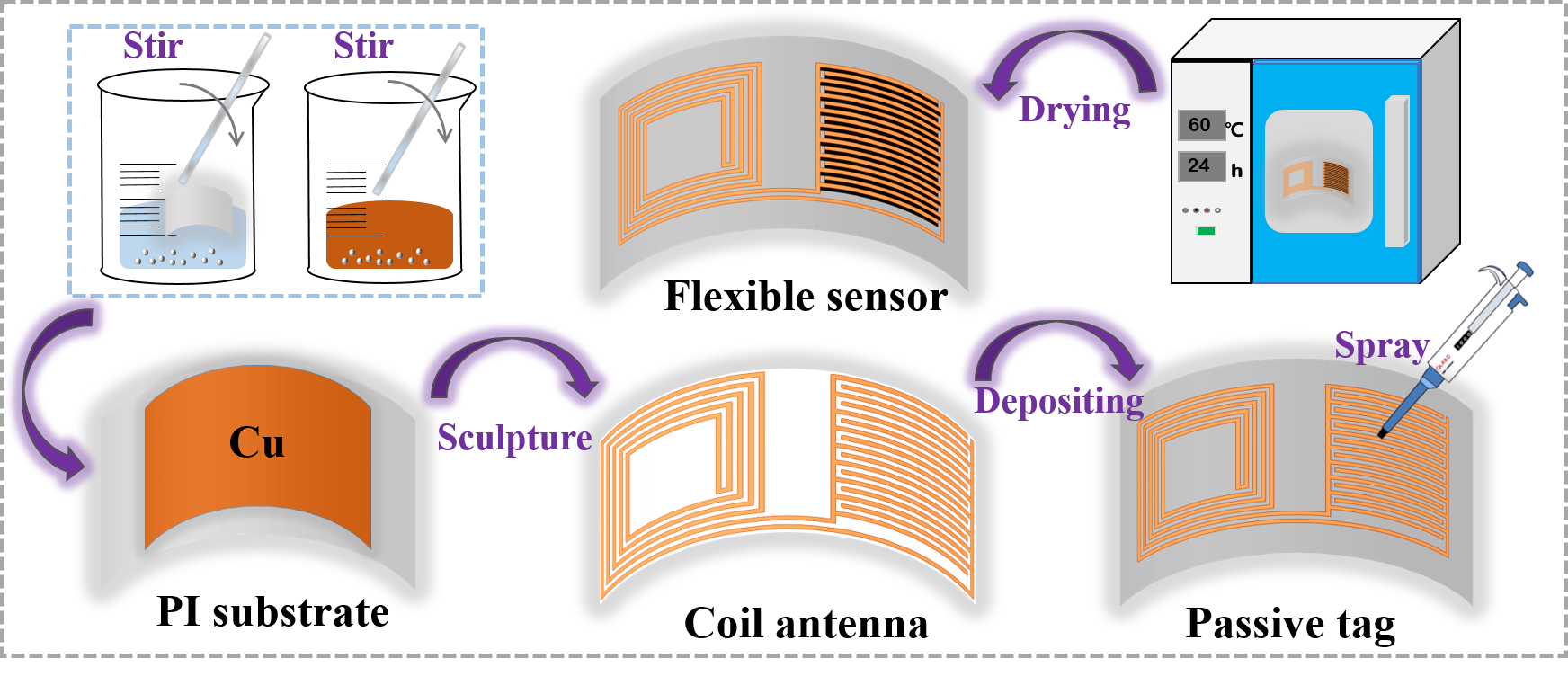


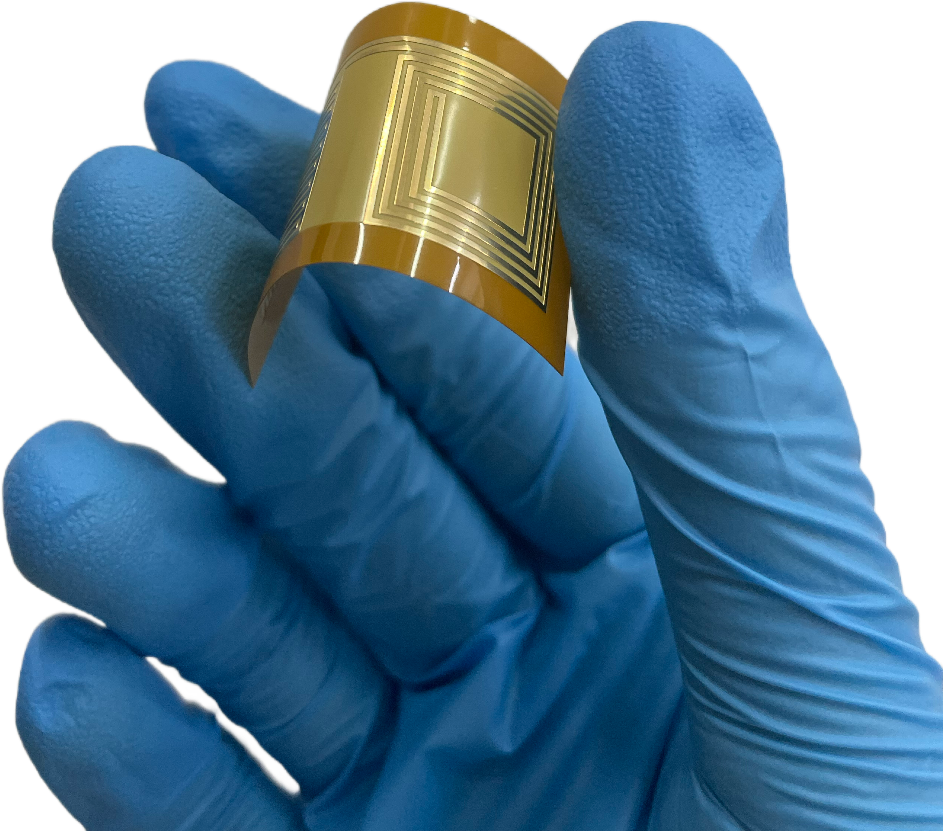


**Figure S20.** Fabrication of flexible chipless RFID sensor tags


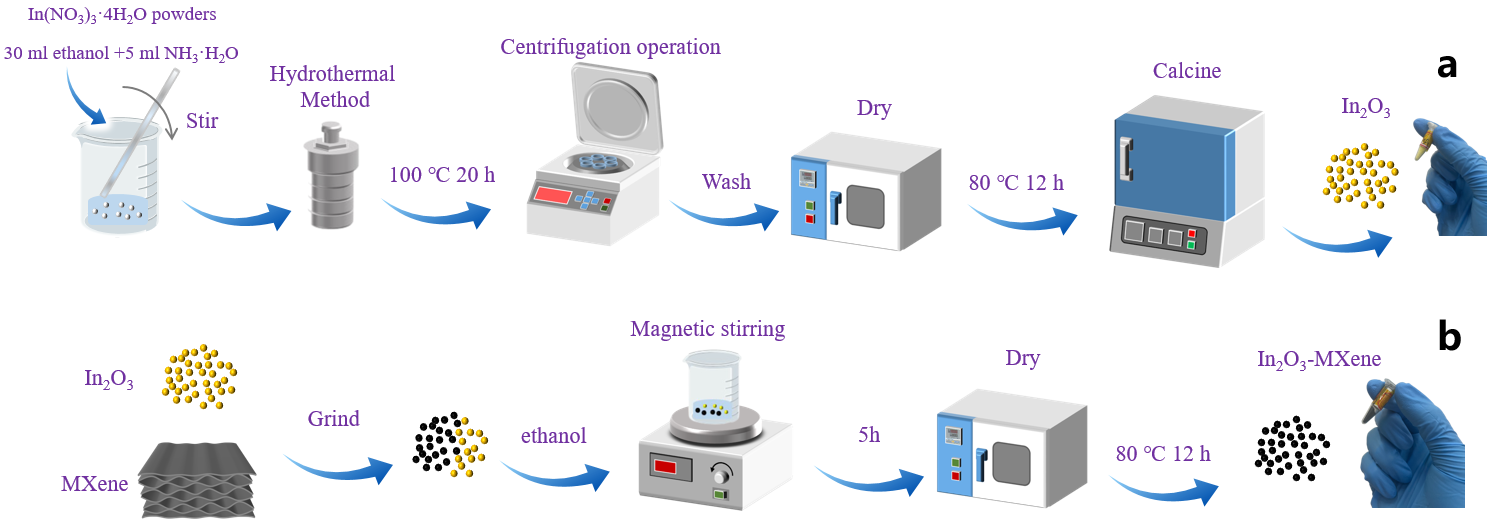


**Figure S21.** a) Preparation of In_2_O_3_ material, b) Preparation of MXene/In_2_O_3_ composite material

**Supplementary Note I**

$$\%Sensorresponse=\frac{\left|\triangle S_{11}\right|}{\left|S_{11},air\right|}\times100,$$

The real part of the sensor’s *S*_11_ parameter in air and ammonia environments, the real part of the *S*_11_ parameter in air, the imaginary part of the *S*_11_ parameter in air and ammonia, and the imaginary part of the *S*_11_ parameter in air are denoted as Re(*S*_11_)_air+gas_, Re(*S*_11_)_air_, Im(*S*_11_)_air+gas_, and Im(*S*_11_)_air_, respectively. The label *S*_11_ value in air is represented by |*S*_11_, air|, the absolute value of the change in the label *S*_11_ coefficient due to ammonia adsorption, which is represented by |Δ*S*_11_|. According to Equation (1), it is directly correlated with the percentage of the sensor response.

**Supplementary Note II**

Mechanism of humidity-enhanced sensing response: Under specific humidity conditions, when ammonia molecules come into contact with the surface of the hybrid sensitive material during detection, solvent-assisted interactions may occur ^[1,2]^, leading to the formation of NH_4_⁺ ions. These ions can further react with the O_2_⁻ ions adsorbed on the sensor membrane surface, generating the corresponding nitrogen oxides. The reaction process is outlined as follows ^[3–5]^:

As illustrated in **Figure S22**, in conjunction with Equations (4)–(6), humidity enhances the ammonia sensing response by providing an alternative reaction pathway. In dry air, NH_3_ directly reacts with the adsorbed oxygen species on the material surface, releasing electrons. In contrast, under humid conditions, NH_3_ first interacts with adsorbed water molecules to form ammonium ions (NH_4_⁺). These positively charged NH_4_⁺ ions subsequently react efficiently with negatively charged adsorbed oxygen species (O_2_⁻), leading to the release of additional electrons. For the MXene/In_2_O_3_ composite dominated by n-type In_2_O_3_, the increased electron back-donation to the conduction band further reduces the depletion layer width and increases the relative dielectric constant (*εᵣ*). According to Equations (4)–(6) in **the** **main text**, this results in a more pronounced increase in the equivalent capacitance of the interdigital electrodes (*C_IDE_*), thereby inducing a larger resonance frequency shift. Based on Equations (1)–(3) in **the Supplementary Information**, the enhancement effect of humidity on the ammonia sensing response is quantitatively evaluated.

Under moderate humidity conditions (RH 30–80%), the synergistic effects of enhanced dielectric constant and interfacial polarization lead to a significantly larger capacitance variation (*ΔC*) induced by NH_3_ adsorption compared to dry conditions, thereby effectively improving the sensing response of the sensor.


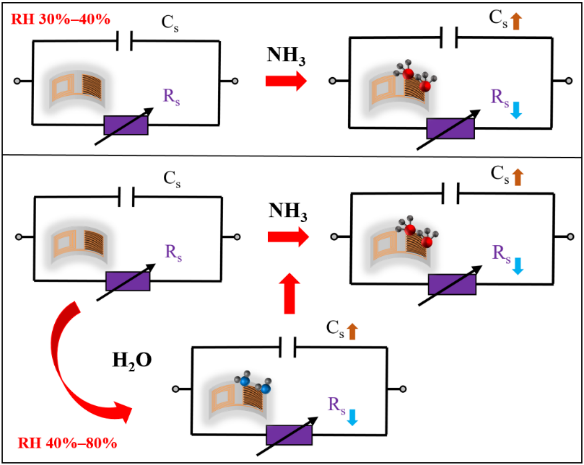


**Figure S22.** Mechanism of humidity-enhanced sensing response

**Supplementary Note III**

**Temperature variation modulates the intrinsic electrical properties of the In_2_O_3_/MXene composite, thereby inducing baseline drift. Specifically, an increase in temperature enhances the thermal excitation of charge carriers, leading to increased electrical conductivity and altered interfacial charge distribution and polarization characteristics. Meanwhile, the surface coverage of adsorbed oxygen species (O_2_^-^) and the Schottky barrier at the In_2_O_3_/MXene interface are dynamically adjusted with temperature, which further modifies the effective dielectric constant in the interdigital electrode region. As a result, the initial equivalent capacitance (*C*_0_) shifts, ultimately manifesting as baseline drift in the resonance frequency under NH_3_-free conditions.**

**At low temperatures (from 25 °C to 5 °C), the activation and adsorption capability of oxygen molecules is weakened, resulting in reduced electron trapping at the surface. This leads to a thinning of the depletion layer and a transition of the material from a relatively “depleted state” to an “electron-enriched state.” Consequently, the interfacial polarization is weakened, leading to a decrease in the effective dielectric constant and capacitance. According to the LC resonance relationship, a decrease in capacitance results in an increase in the resonance frequency, corresponding to a positive baseline shift (Figure S23).**

**In contrast, at elevated temperatures (from 25 °C to 45 °C), although the thermal motion of gas molecules is enhanced, the activation of adsorbed oxygen species and electron trapping dominate within this temperature range. This results in a deepened depletion layer and a more pronounced electron-depleted state, accompanied by enhanced interfacial polarization. Consequently, the effective dielectric constant and capacitance increase, leading to a decrease in the resonance frequency according to the LC resonance principle, i.e., a negative baseline shift. In addition, to systematically evaluate the effect of temperature fluctuations on the baseline stability of the sensor, baseline drift measurements were conducted over a temperature range of 5–45 °C, which is typical for agricultural product storage and transportation, using 25 °C as the reference. As shown in the figure, when the temperature decreases to 5 °C, the baseline resonant frequency increases by approximately 50 KHz relative to that at 25 °C, whereas an increase in temperature to 45 °C results in a decrease of about 80 KHz. These results indicate that variations in ambient temperature can induce significant baseline frequency drift, thereby affecting the accuracy of ammonia detection. In subsequent experiments and data processing, the influence of environmental factors on the sensor baseline was fully considered, and the frequency shifts induced by such factors were systematically corrected to ensure the reliability of the sensing results.**

$$C_{\text{IDE}}=C_{0}+\Delta C_{\text{NH}_{3}}$$

**
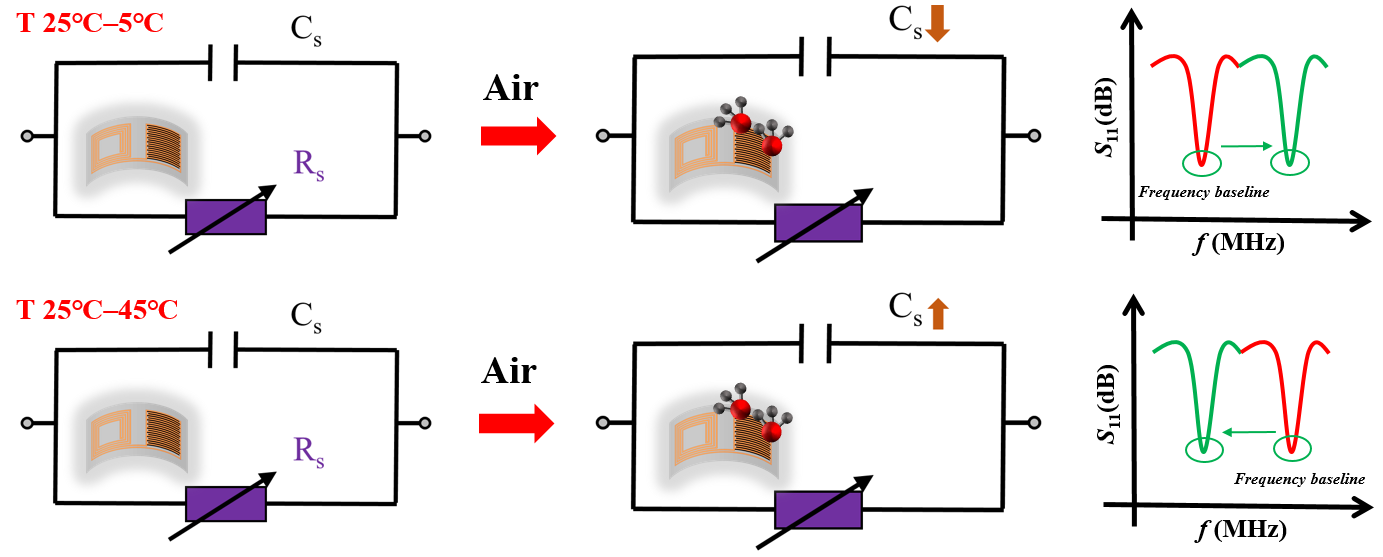
**

**Figure S23.** Mechanism of temperature fluctuation–induced sensing response

**Supplementary Note IV**

**The total volatile basic nitrogen (TVB-N) content of fresh fish and beef samples was determined using the semimicro distillation method. At 4 h intervals, one fish sample and one beef sample were randomly selected from a sealed storage container. For fish, the skin and bones were removed, and muscle tissue was collected from the abdominal region; for beef, samples were taken from the tenderloin region. The samples were cut into approximately 0.5 cm cubes using a sterile surgical scalpel. Exactly 4.00 g of fish or beef was weighed into a 50 mL beaker, followed by the addition of 30.00 mL of deionized water. The mixture was homogenized using a tissue homogenizer (JRJ300-I, Changzhou, China) at 12,000 rpm for 1 min until a uniform homogenate without visible particles was obtained. The homogenate was then transferred into a 50 mL centrifuge tube and extracted in a thermostatic shaker at 90 rpm for 40 min. Subsequently, the mixture was centrifuged at 4,500 rpm for 8 min at 4 °C. The supernatant was collected for further analysis; if an oil layer was present on the surface, it was carefully removed using a pipette. The distillation was performed using a semiautomatic Kjeldahl nitrogen analyzer (KDN-103F, Shanghai, China). A 6.00 mL aliquot of the supernatant was mixed with 4.00 mL of magnesium oxide solution (12 g/L) and injected into the distillation chamber. For the blank control, an equal volume of distilled water was used instead of the sample solution, while all the other conditions were kept constant. The distillation time was set to 7 min, and the distillate was collected in an Erlenmeyer flask containing 20.00 mL of boric acid solution (12 g/L) with three drops of mixed methyl red–bromocresol green indicator. The collected distillate was titrated with 0.01 mol/L standard hydrochloric acid solution until the color changed from blue‒green to light purplish red, and the volume of acid consumed was recorded. Each sample was analyzed in triplicate, while the blank was measured twice. The final results were obtained by averaging the measurements and subtracting the blank consumption. The TVB-N content was calculated and expressed as mg per 100 g of fish or beef. Calculation: The TVB-N value was calculated on the basis of the volume of hydrochloric acid consumed using Equation (7), and the results are expressed as milligrams of nitrogen per 100 g of fish or beef:**

$$\text{TVB-N}=(mg/100g)=\{[(V_{s}-V_{b})\times C\times14]/[m\times(5/V)]\}\times100$$

**where *Vs* and *V_b_* denote the volumes (mL) of hydrochloric acid consumed for titration of the sample and the blank control, respectively; *C* is the concentration of hydrochloric acid (0.01 mol/L); m is the mass of the fresh meat sample (3 g); and *V* is the total volume of the fresh meat homogenate (30 mL).** Seven groups of samples were stored at room temperature and analyzed at 4, 8, 12, and 16 h to evaluate the spoilage status of the fish and beef. Each group is represented by the mean value of three independent measurements.

**Supplementary Table I**: Analysis of *S*_11_ amplitude corresponding to medium transmission loss

| Plastic | | | Cardboard | | | Foam | | | Glass | | |
| --- | --- | --- | --- | --- | --- | --- | --- | --- | --- | --- | --- |
| TL | MV(dB) | EV(dB) | TL | MV(dB) | EV(dB) | TL | MV(dB) | EV(dB) | TL | MV(dB) | EV(dB) |
| 6.41 | -31.17 | 0.25 | 9.12 | -30.03 | 0.29 | 10.47 | -26.52 | 0.26 | 16.27 | -24.47 | 0.21 |
| 9.91 | -28.96 | 0.35 | 12.72 | -28.53 | 0.21 | 14.47 | -24.39 | 0.16 | 21.39 | -22.26 | 0.15 |
| 10.01 | -28.39 | 0.26 | 13.4 | -28.07 | 0.24 | 15.66 | -24.23 | 0.12 | 22.07 | -22.21 | 0.2 |
| 14.01 | -26.73 | 0.17 | 17.52 | -26.12 | 0.12 | 19.06 | -23.22 | 0.22 | 25.59 | -21.36 | 0.22 |
| 14.51 | -26.33 | 0.2 | 17.6 | -25.89 | 0.11 | 19.27 | -22.5 | 0.16 | 27.07 | -20.32 | 0.21 |
| 16.06 | -25.85 | 0.16 | 18.92 | -25.33 | 0.09 | 20.36 | -22.29 | 0.17 | 27.99 | -20.14 | 0.34 |
| 19.11 | -24.36 | 0.14 | 21.6 | -24.85 | 0.11 | 23.56 | -20.7 | 0.05 | 30.35 | -19.49 | 0.27 |
| 19.44 | -24.01 | 0.22 | 22.92 | -23,47 | 0.2 | 24.06 | -20.36 | 0.22 | 31.59 | -19.14 | 0.23 |
| 19.66 | -23.79 | 0.21 | 23.8 | -23 | 0.13 | 25.26 | -20.07 | 0.23 | 32.19 | -19.01 | 0.27 |
| 22.64 | -22.32 | 0.21 | 27.12 | -21.52 | 0.1 | 28.76 | -18.34 | 0.22 | 35.19 | -17.46 | 0.21 |
| 23.86 | -21.5 | 0.26 | 27.8 | -21.31 | 0.14 | 28.78 | -18.35 | 0.13 | 35.95 | -17.04 | 0.17 |
| 24.63 | -21.24 | 0.01 | 30.13 | -20.19 | 0.22 | 30.4 | -18.02 | 0.19 | 36.1 | -16.61 | 0.08 |
| 27.84 | -19.24 | 0.07 | 30.8 | -19.86 | 0.15 | 34 | -16.22 | 0.11 | 40.35 | -14.71 | 0.11 |
| 28.83 | -19.03 | 0.16 | 33.73 | -18.96 | 0.24 | 34.38 | -16.07 | 0.18 | 40.3 | -14.6 | 0.23 |
| 33.23 | -17.53 | 0.04 | 37.53 | -16.81 | 0.15 | 38.8 | -14.01 | 0.12 | 45.2 | -12.16 | 0.17 |

Note: *N*=5 means the measurement time is 5, The error value is the standard deviation calculated with five independent tests. MV: Mean Value, EV: Error Value

**Supplementary Table II**: Error analysis of a nonlinear ammonia quantification model based on frequency responses associated with fish freshness deterioration

| NH_3_ Concentration | *f*_(Measured)_ | *f*_(Linear)_ | Relative error |
| --- | --- | --- | --- |
| 3 ppm | 13.43 MHz | 13.39 MHz | 0.3% |
| 4 ppm | 13.29 MHz | 13.26 MHz | 0.2% |
| 5 ppm | 13.18 MHz | 13.14 MHz | 0.3% |
| 6 ppm | 13.11 MHz | 13.05 MHz | 0.5% |
| 7 ppm | 13.06 MHz | 12.97 MHz | 0.7% |
| 8 ppm | 12.98 MHz | 12.9 MHz | 0.6% |
| 9 ppm | 12.94 MHz | 12.84 MHz | 0.8% |
| 10 ppm | 12.91 MHz | 12.79 MHz | 0.9% |

**Supplementary Table III**: Error analysis of a nonlinear ammonia quantification model based on frequency responses associated with beef freshness deterioration

| NH_3_ Concentration | *f*_(Measured)_ | *f*_(Linear)_ | Relative error |
| --- | --- | --- | --- |
| 3 ppm | 13.45 MHz | 13.4 MHz | 0.4% |
| 4 ppm | 13.31 MHz | 13.26 MHz | 0.4% |
| 5 ppm | 13.19 MHz | 13.15 MHz | 0.3% |
| 6 ppm | 13.11 MHz | 13.06 MHz | 0.4% |
| 7 ppm | 13.05 MHz | 12.98 MHz | 0.5% |
| 8 ppm | 13.01 MHz | 12.92 MHz | 0.7% |
| 9 ppm | 12.96 MHz | 12.86 MHz | 0.8% |
| 10 ppm | 12.93 MHz | 12.81 MHz | 0.9% |

Ref:

[1] L. Liu, T. Fei, X. Guan, et al. “Room temperature ammonia gas sensor based on ionic conductive biomass hydrogels,” *Sens. Actuators B Chem.* 320, (2020): 128318. *https://doi.org/10.1016/j.snb.2020.128318*

[2] S. Wang, Y. Fu, T. Wang, et al. “Fabrication of robust and cost-efficient Hoffmann-type MOF sensors for room-temperature ammonia detection,” *Nat. Commun.* 14 (2023): 7261. *https://doi.org/10.1038/s41467-023-42959-z*

[3] M. Liu M, J. Wang, P. Song, et al. “Metal–organic frameworks-derived In₂O₃ microtubes/Ti_3_C_2_T_x_ MXene composites for NH_3_ detection at room temperature,” *Sens. Actuators B Chem.* 361 (2022): 131755. [*https://doi.org/10.1016/j.snb.2022.131755*](https://doi.org/10.1016/j.snb.2022.131755)

[4] N. Sharma, N. Sharma, P. Srinivasan, et al. “Heptazine-based organic framework as a chemiresistive sensor for ammonia detection at room temperature,” *J. Mater. Chem. A* 6 (2018): 18389–18395.

[5] X. Li, X. Li, Z. Li, et al. “WS_2_ nanoflakes-based selective ammonia sensors at room temperature,” *Sens. Actuators B Chem.* 240 (2017): 273–277. [*https://doi.org/10.1016/j.snb.2016.08.163*](https://doi.org/10.1016/j.snb.2016.08.163)
